# Highly efficient nickel (II) removal by sewage sludge biochar supported α-Fe_2_O_3_ and α-FeOOH: Sorption characteristics and mechanisms

**DOI:** 10.1371/journal.pone.0218114

**Published:** 2019-06-12

**Authors:** Lie Yang, Liuyang He, Jianming Xue, Li Wu, Yongfei Ma, Hong Li, Pai Peng, Ming Li, Zulin Zhang

**Affiliations:** 1 Hubei Key Laboratory of Mineral Resources Processing and Environment, School of Resources and Environmental Engineering, Wuhan University of Technology, Wuhan, PR China; 2 New Zealand Forest Research Institute Limited (Scion), Forest System, Christchurch, New Zealand; 3 The James Hutton Institute, Craigiebuckler, Aberdeen, United Kingdom; RMIT University, AUSTRALIA

## Abstract

A novel approach was employed to load α-Fe_2_O_3_ and α-FeOOH onto sewage sludge biochar (SBC) with the purpose of efficient nickel (Ni) removal. A high Ni(II) adsorption capacity of 35.50 mg·g^-1^ in 100 ppm Ni(II) solution with 10 mg modified sewage sludge biochar (MSBC) was achieved. The adsorption kinetic and isotherm were fitted well by the pseudo-second-order model and the Langmuir model, respectively. The optimal pH was found around a neutral pH of 7. The adsorption mechanisms of Ni(II) onto MSBC were described as the synergistic effects of electrostatic attraction, ion exchange, inner-sphere complexation and co-precipitation. The initial rapid adsorption phenomenon could be attributed to electrostatic attraction and ion exchange, and then inner-sphere complexation and co-precipitation acted as a crucial role in the following step. The remarkable performance of MSBC provides an effective waste utilization approach to simultaneous sewage sludge recycle and Ni removal from aqueous solution.

## Introduction

Nickel (II) ion (Ni (II)), a toxic heavy metal ion, is discharged into natural waters mainly from various chemical industries, including textile dyeing, electroplating, and metal finishing [1]. Once the concentration of Ni(II) increases to a limit in the water bodies, there is a potential threat to food security and human health. An investigation was conducted in eastern Taiwan to evaluate the health risks of Ni in rice, and much higher Ni concentrations were observed in rice grain from Ni contaminated fields [2]. Furthermore, the incidence of oral cancer was found correlated positively with levels of Ni in farm soils based on a compulsory national health insurance program in Taiwan [3]. Soil contamination with Ni was frequently caused by discharge of Ni wastewater without appropriate treatment. Therefore, effective and economical technologies for the Ni removal in wastewater are urgently needed.

So far, numerous attempts were made to eliminate Ni contamination, including chemical precipitation [4], phytoremediation [5], redox [6] and adsorption [7, 8]. Among them, adsorption is an effective technology for Ni removal and potentially for Ni recycle based on desorption [9]. In recent years, adsorbents derived from various wastes including agricultural and municipal wastes [10, 11], have attracted great attention due to the preference of waste disposal and recycle. Among diverse wastes, sewage sludge is a potential source for adsorbents due to its huge annual production from municipal wastewater treatment plants. However, raw sewage sludge is water swelling and generally contains various pollutants [12, 13]. Therefore, appropriate treatment is necessary to make this common solid waste to be a stable and safe adsorbent. There have been several studies focused on sewage sludge treatment especially for biochar production [14–16]. Pyrolysis of sewage sludge offers the advantage of releasing less flue gas that has to be managed as compared to incineration of sewage sludge and biochar can be obtained as potential adsorbents from the pyrolysis process [17]. Additionally, engineered (smart) biochar production from sewage sludge and willow has been attempted based on various carrying gases and mixture ratios of sludge and willow [18]. It is common that the biochar with appropriate modification presents higher surface areas and larger adsorption capacities than the raw wastes [19]. There has been several studies concerning Ni adsorption of biochar derived from bio-wastes except sewage sludge. For the sludge-derived biochar application, the research objective and modified methods of the relevant studies were mostly suitable for lead [14], cadmium [16], and some organic pollutants [20] rather than Ni.

In recent years, special attention was paid to α-Fe_2_O_3_ [21, 22] and α-FeOOH [23, 24] modification of adsorbents due to the outstanding improvement of the adsorption capacity. O-H was confirmed to be beneficial for copper adsorption [23] and it was found that 4–10 iron-bonded hydroxyl groups per nm^2^ existed on the surface of α-Fe_2_O_3_ [25]. In addition, the interaction between α-FeOOH and Cu^2+^ was found possibly via the formation of Fe-O-Cu complexes [23], indicating that heavy metal ions could react with Fe-OH. The above characteristics of α-Fe_2_O_3_ and α-FeOOH are potential for enhancing the Ni adsorption from aqueous solution like Cu^2+^. Until now, no systematic study concerning the adsorption performance of biochar with α-Fe_2_O_3_ and α-FeOOH in Ni (II) removal from aquatic environment has been published yet. To develop sewage sludge biochar for efficient Ni removal, we prepared sludge biochar (SBC) and then modified sludge biochar (MSBC) with α-Fe_2_O_3_ and α-FeOOH for Ni adsorption in aqueous solution. BET, XRD, SEM-EDS and FTIR were employed to characterize the prepared biochar. Dosages and pH were tested for the influences of adsorption parameters. In addition to the adsorbent regeneration, sorption kinetics and isotherms were assessed for the adsorption mechanisms in aqueous solution. This study could provide an efficient approach for simultaneously Ni removal and sludge application.

## Material and methods

### Materials

Iron(III) chloride hexahydrate (FeCl_3_·6H_2_O, ≥99%), iron(II) sulfate heptahydrate (FeSO_4_·7H_2_O, ≥99%), Ni nitrate(≥99%), sodium hydroxide (≥96%) and hydrochloric acid (36%) were purchased from Sinopharm Chemical Reagent Co., Ltd. (Shanghai, China). All reagents used in the experiments were of analytical grade and solutions were prepared with deionized water. The sewage sludge was provided by Tangxunhu Municipal Wastewater Plant in Wuhan, China. The obtained sludge was oven dried at 60 °C for 24 h and then went through slow pyrolysis at 500 °C with a heating rate of 10 °C·min^-1^ for 2 h to produce the sludge biochar (SBC). The obtained SBC was milled and then sieved (<0.074 mm) for further experiments.

### Preparation of modified sludge biochar

The modified sludge biochar (MSBC) was fabricated based on SBC with the following procedure. FeCl_3_·6H_2_O (40 g) and FeSO_4_·7H_2_O (22.2 g) were dissolved in 1200 mL deionized water and then 30 g SBC was added into the solution. The mixture was stirred at room temperature (25 ~ 30 °C) for 30 min. Subsequently, 10 M NaOH solution was added into the mixture until the pH reached 10 and stirred for 2 h. After precipitation, the mixture was filtrated and the residual was washed with deionized water and ethanol several times and dried at 70 °C for 24 h. The obtained MSBC powder (0.074 mm) was kept for further experiments.

### Characterizations

The surface area, pore width and pore volume of SBC and MSBC were measured with a Micromeritics ASAP 2020 analyzer (USA). The crystalline structure analysis was conducted with D8 X-ray diffraction spectra (Bruker, Germany). Fourier-transform infrared spectroscopy (FTIR) spectra were obtained with IRprestige-21 (Shimadzu, Japan) using the KBr pellet method and examined in the 4000–400 cm^-1^ region. The software named OMNIC was applied to analyze the FTIR spectra. The surface morphologies of the powder samples were analyzed by a JSM-7100F SEM (JEOL, Japan).

### Batch experiments

To measure the adsorption capacities of SBC and MSBC adsorbents, 10–100 mg of these adsorbents were added to 50 ml of Ni(II) solution (100 ppm) at 25 °C for 24 h. Various pH values (2–7) were set to find the optimal pH for Ni(II) removal. The pH was adjusted to the desired values by using 0.01 M NaOH and HCl. Kinetic experiments were conducted by adding 50 mg MSBC adsorbent to 50 ml of Ni(II) solution (100 ppm) at 25 °C for various time intervals (2, 5, 10, 30 min and 1, 4, 8, 12, 18 h). The vials were sealed and shaken in a constant temperature shaker for 18 h to obtain the adsorption equilibrium. Correspondingly, an adsorption isotherm study was conducted by adding 50 mg of MSBC with 50 ml of Ni(II) solution (20–1000 ppm) at 25 °C. Finally, the desorption experiment was carried out using Na_2_EDTA solution. The Ni loaded MSBC was soaked in 0.1 M Na_2_EDTA solution and shaken for 2 h. And then, the Ni-desorbed MSBC was employed for further recycle experiments. The concentrations of Ni(II) solutions were measured by a 700P atomic absorption spectroscopy (Analytik Jena, Germany). The amount of adsorbed Ni(II) was calculated according to a previous study [14]. SPSS 19.0 and Microsoft Excel 2010 were used for data analysis in the kinetics and isotherm study, and Origin 8.0 was applied for graphing.

## Results and discussion

### Characterizations

The surface area, pore width and pore volume results of SBC and MSBC are presented in Table 1. The pore volume and pore width of SBC were larger than those of MSBC, while MSBC had a larger surface area (S_BET_). As reported in previous studies, S_BET_ is critical for the adsorption of heavy metals including Ni [14, 26–28]. In this study, the widely distributed iron compounds on the surface of MSBC significantly increased S_BET_ of MSBC (34.99 m^2^·g^-1^). The higher S_BET_ of MSBC was of significant benefits to the adsorption capacity for Ni removal (Table 1). The reduction of the pore volume and pore width during the fabrication of MSBC could be attributed to the occupation of iron compounds into the pores of the biochar.

**Table 1:** Pore distribution properties and Ni adsorption capacity of SBC and MSBC.

Biochar	pH	S_BET_ (m^2^·g^-1^)	Pore volume ^a^ (cm^3^·g^-1^)	Pore width (Å)	Adsorption capacity^b^ (mg/g)
SBC	8.12	24.21	0.0837	138.33	20.38
MSBC	8.73	34.99	0.0802	91.68	35.50

^a^ Pore Volume determined at P/Po = 0.99;

^b^ Adsorption average pore width (4V/A by BET).

The XRD patterns of crystalline structures and composition of SBC and MSBC are shown in S1 Fig. Sharp reflection peaks at 20.86°, 26.30°, 36.55°, 42.14°, 50.15°, 60.00° and 68.08° in SBC and MSBC could be indexed as the planes of SiO_2_ (PDF#65–0466), which were in line with the data in a previous study [29]. Therefore, SiO_2_ could be reasonably considered as the dominating crystalline phase in both of SBC and MSBC. Furthermore, several peaks at 27.91°, 34.79°, 39.12°, 40.25°, 45.90°, 54.95° and 19.80°, 36.50°, 55.80°, 60.00° for MSBC revealed the formation of α-Fe_2_O_3_ (PDF#16–0653) and α-FeOOH (PDF#26–0792), respectively. The formation processes could be described by reactions (1)–(5). These reactions were confirmed by a previous study by Xiong and Zhou [30]. It has been demonstrated that Fe(OH)_3_ can produce the mixture of FeOOH and α-Fe_2_O_3_ at the range of 40–80 °C, but only α-Fe_2_O_3_ at above 80°C [30]. The drying temperature was set at 70°C in this study, which could probably explain the observed crystalline phases.

$${\text{FeCl}}_{3}+3\text{NaOH}\to \text{Fe}{\left(\text{OH}\right)}_{3}+3\text{NaCl}$$ (1)

$$2\text{Fe}{\left(\text{OH}\right)}_{3}\to \alpha -{\text{Fe}}_{2}{\text{O}}_{3}+3{\text{H}}_{2}\text{O}$$ (2)

$${\text{FeSO}}_{4}+2\text{NaOH}\to \text{Fe}{\left(\text{OH}\right)}_{2}+{\text{Na}}_{2}\text{SO}4$$ (3)

$$4\text{Fe}{\left(\text{OH}\right)}_{2}+2{\text{H}}_{2}\text{O}+{\text{O}}_{2}\to 4\text{Fe}{\left(\text{OH}\right)}_{3}$$ (4)

$$\text{Fe}{\left(\text{OH}\right)}_{3}\to \alpha -\text{FeOOH}+{\text{H}}_{2}\text{O}$$ (5)

**Fig 1:**
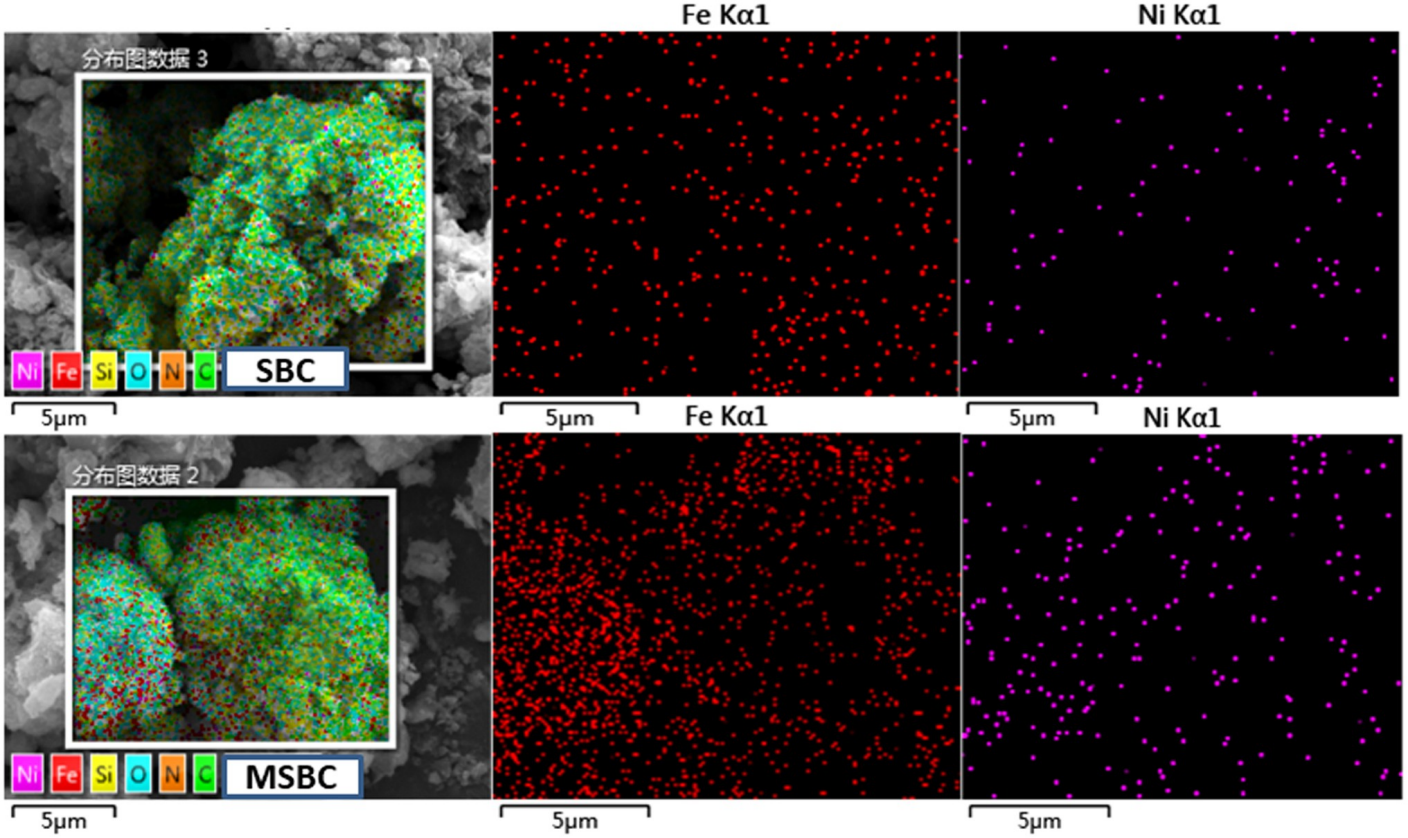
SEM-EDX analysis on the surface of SBC and MSBC after Ni adsorption.

The external morphology of SBC and MSBC at both 5000 and 10000 magnifications are presented in S2 Fig. Obvious pore structures were observed on the surfaces of both SBC and MSBC. However, the conspicuous particles were only distributed on the surface of MSBC (S2(c) and S2(d) Fig) but not SBC. These particles could be confirmed as iron oxides based on the above XRD analysis and SEM-EDS images in Fig 1. In addition, significant reduction of pore width on the surface of MSBC can be observed due to the occupation of iron compounds into the pores of adsorbents. This phenomenon was in agreement with the results of BET analysis (Table 1). The SEM-EDS images are presented in Fig 1, which revealed the characteristics of iron and Ni distribution on the surface of SBC and MSBC. It was observed that iron compounds distributed on the surface of SBC and MSBC and the iron percentage of MSBC was distinctly higher than that of SBC due to the fabrication process. It was worth noting that the Ni adsorption amount of MSBC was evidently higher compared with SBC (Fig 1). Furthermore, the Ni distribution locations appeared nearly consistent with the locations of the iron distribution. This provided evidence for the positive effects of α-Fe_2_O_3_ and α-FeOOH micro particles on Ni adsorption.

The FTIR spectra of SBC and MSBC before and after Ni adsorption are showed in S3 and S4 Figs. As for SBC, the spectrum before sorption shows the adsorption bonds at 3379 cm^-1^, 1620 cm^-1^ and 1037 cm^-1^ for O-H, C = C and C-O, respectively [14, 31]. The band at 1080 cm^−1^ could be the P-O stretching vibrations of PO_4_^3−^ [32, 33]. The bands at 797 cm^-1^ and 474 cm^-1^ were attributed to the deformation and bending modes of the Si-O bond [34, 35]. The slight peak at 551 cm^-1^ could be attributed to the Fe-O stretching vibration in α-Fe_2_O_3_ [34, 36], which indicated that there was original α-Fe_2_O_3_ in SBC. The spectra of MSBC was similar to that of SBC for strong bonds. An additional signal at 602 cm^-1^ could be assigned to the Fe-OH bending vibrations [23, 37], which was the solid evidence for the existence of α-FeOOH. These results further supported the findings obtained from XRD analysis.

### Adsorption study

#### Adsorption capacity

**Fig 2:**
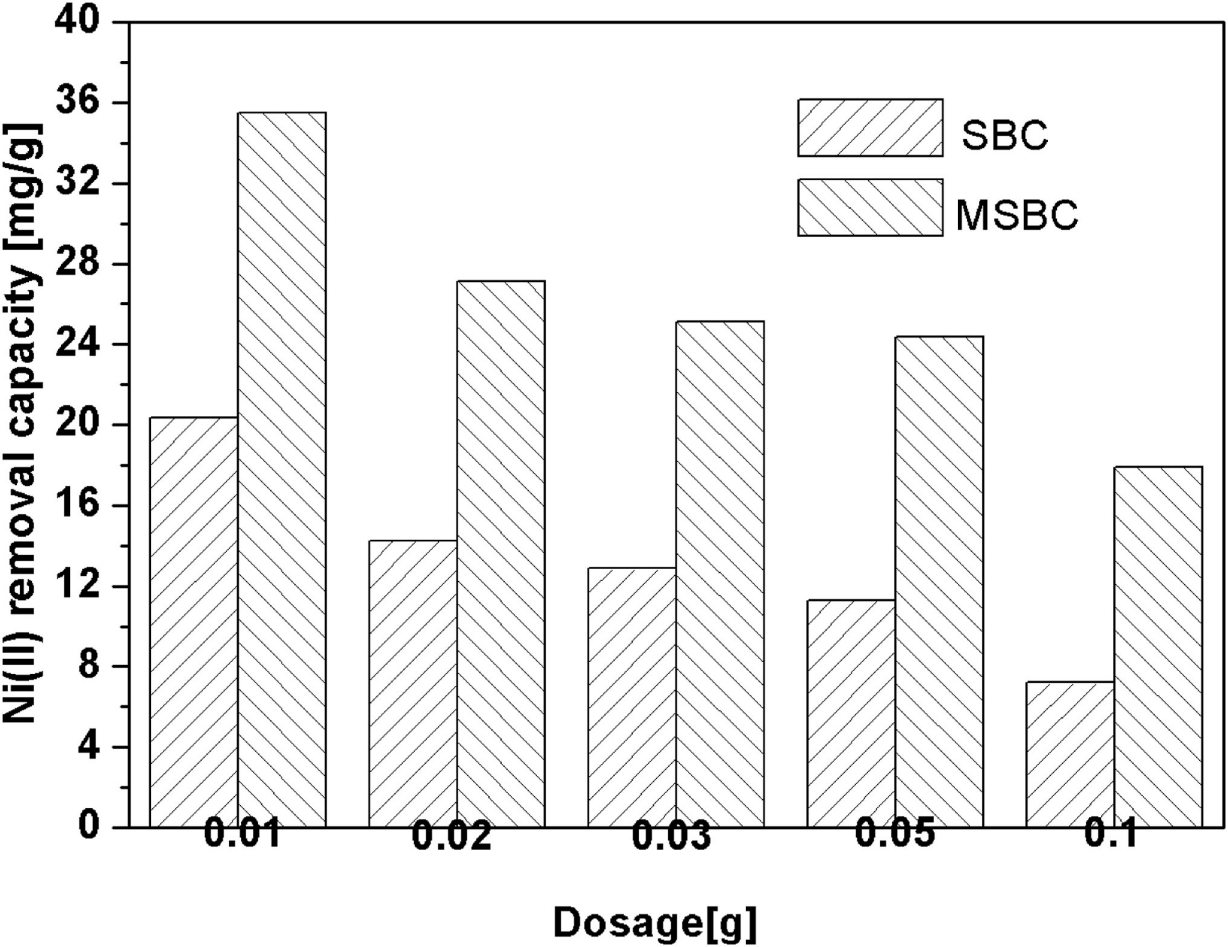
Comparison of SBC and MSBC adsorption capacities with various doses for Ni removal. Reaction conditions: Initial concentration of Ni(II) 100 ppm at pH 7, 50 ml of solution, 1~100 mg of biochar, 24 h, 25 °C.

Undoubtedly, adsorption capacity is a crucial parameter for adsorbents. Various studies have been conducted to develop novel adsorbents with efficient adsorption performance [38–40]. The comparison of SBC and MSBC adsorption capacities with various dosages for Ni removal is presented in Fig 2. It was evident that the adsorption capacity of MSBC was significantly higher than that of SBC at all dosage levels, which indicated that the fabrication procedure was effective for promotion of Ni adsorption. The maximum capacity of 35.50 mg·g^-1^ was achieved at the dosage of 0.01 g MSBC and decreased with the dose growth of adsorbent. Other biochar adsorbents were compared with SBC and MSBC for Ni removal (Table 2). It was obvious that the adsorption capacity of MSBC exceeded that of most biochar adsorbents as listed in Table 2. This remarkable performance could be due to multiple mechanisms between Ni ions and MSBC, which were discussed in Section 3.4.

**Table 2:** Comparison of the maximum adsorption capacity of Ni on SBC, MSBC and various pyrolysis biochars.

Biochar	Adsorption capacity of Ni (mg/g)	References
Palm seed-based biochar	28.00	[27]
Residues biochar	30.88	[41]
Chicken manure biochar	10.90	[42]
Wheat straw pellets biochar	22.89	[35]
Corncobs biochar	15.40	[8]
Corncobs biochar	29.06	[48]
Hizikia fusiformis biochar	10.39	[49]
Chrysanthemum indicum biochar	29.44	[50]
Sugarcane bagasse biochar (milled)	38.15	[51]
Sugarcane bagasse biochar (unmilled)	6.46	[51]
SBC	20.38	This study
MSBC	35.50	This study

#### Effect of pH

**Fig 3:**
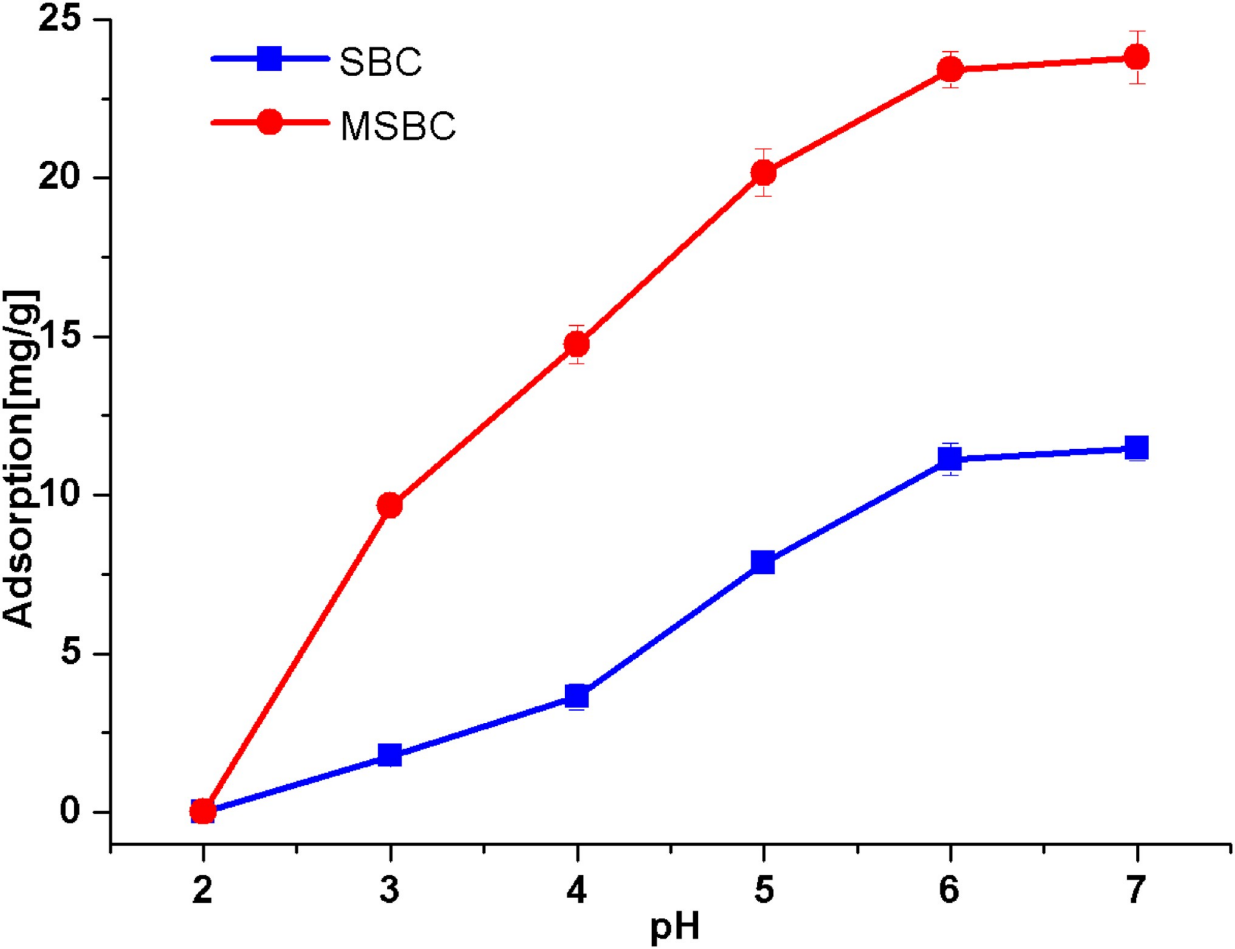
The influence of initial pH on adsorption of Ni(II) solution. Reaction conditions: Initial concentration of Ni(II) 100 ppm at pH 2~7, 25 ml of solution, 50 mg of SBC and MSBC, 24 h, 25 °C.

The increase from pH 2 to pH 7 caused the increase in the adsorption capacity of Ni(II) by both SBC and MSBC (Fig 3). The variations could be attributed to the differences of the surface groups of SBC and MSBC under different solution pH [41]. At a low pH (pH = 2), nearly no Ni(II) adsorption was observed due to the protonation of surface groups by H^+^ ions derived from the solution [41]. It was believed that the deprotonation of the functional groups would occur resulting in less competition of the metal ions with protons for the same binding sites, thus leading to increases in the capture of metal ions on the surface of the biochar [42]. Therefore, Ni(II) ions could be bonded to the surface of the biochar with the increase of pH (pH = 2~5). In the pH range of 2~5, the Ni existed in the form of Ni(OH)^+^, resulting lower electrostatic repulsion between the ion and the surface of the biochar [41]. A previous zeta potential analysis indicated that the zeta potential values of sewage sludge biochar were below -30 mV at pH 4~8 [14]. Thus strong electrostatic attraction would occur between electronegative biochar surface and cationic Ni^2+^ and Ni(OH)^+^. According to Fig 3, slight increases were observed at pH>6, probably due to the precipitation of Ni(II) ions on the surface of biochar in the form of hydroxides. This phenomenon was also observed in a previous Ni(II) adsorption study [35]. The pH_pzc_ values of biochar were found within the range of 7.3–7.8 [35]. Overall, neutral initial pH 7 is beneficial for wastewater treatment. Therefore, pH 7 is the optimal value for Ni(II) adsorption in this study.

#### Sorption kinetics

**Fig 4:**
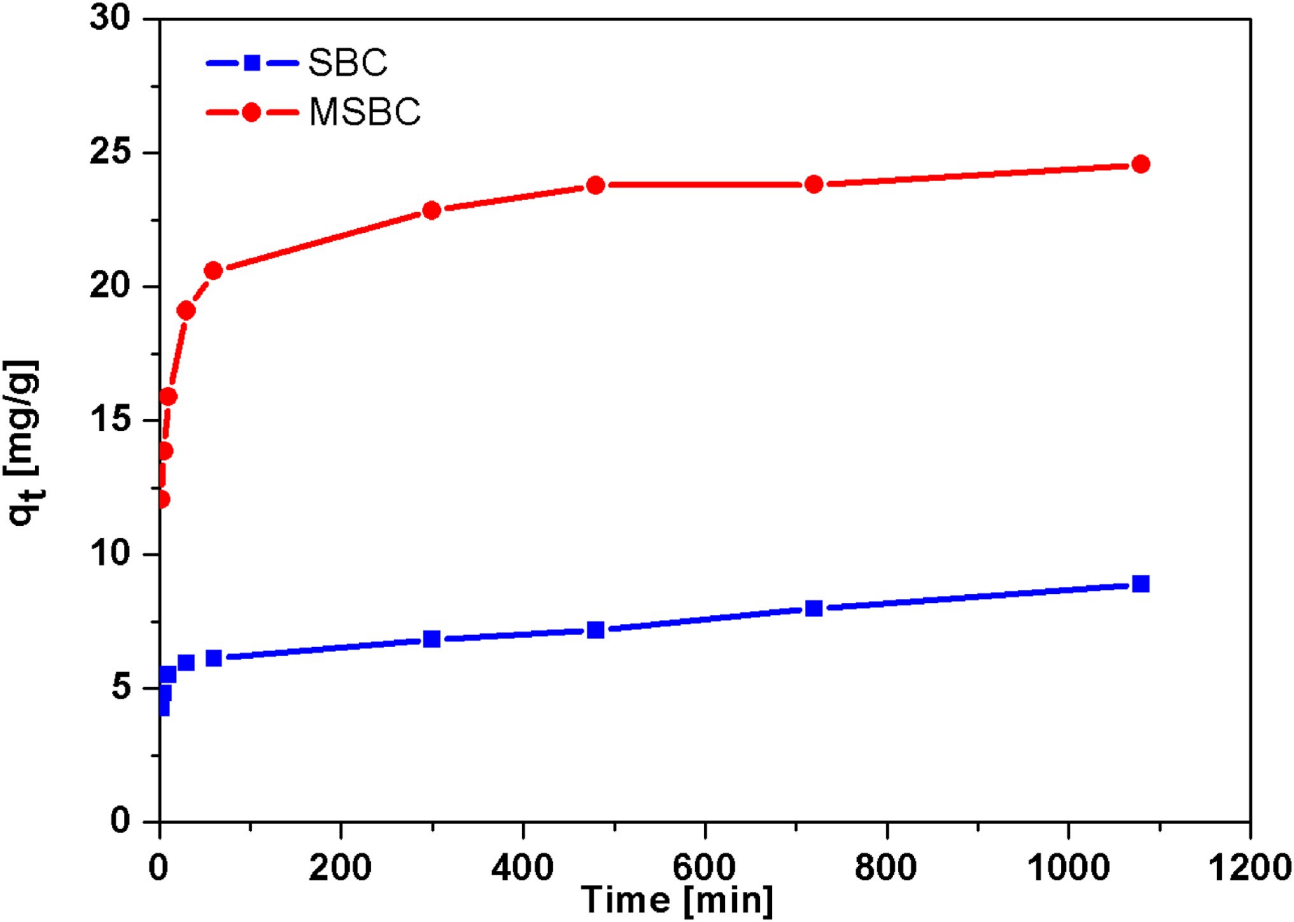
The kinetic of Ni(II) adsorption onto SBC and MSBC. Reaction conditions: Initial concentration of Ni(II) 100 ppm at pH 7, 50 ml of solution, 50 mg of MSBC, 18 h, 25 °C.

The adsorption kinetics of MSBC on Ni(II) removal are shown in Fig 4. The parameter of q_t_ represents the adsorbed amount of Ni(II) at a certain time. The adsorption amount of Ni(II) gradually increased and reached an equilibrium level within 18 hours. It was noticed that the adsorption of Ni(II) achieved nearly 50% of the maximum adsorption capacity at 100 ppm concentration of Ni(II) within 2 min. Several previous studies reported the similar adsorption process when biochar was utilized to adsorb heavy metal ions [35]. The rapid adsorption phenomenon could be attributed to electrostatic attraction and ion exchange, which took a few minutes to reach equilibrium based on available literatures [43]. The pseudo-first-order and pseudo-second-order models were employed to investigate the adsorption kinetic as shown in the equation below:

$$\text{ln}\left({\text{q}}_{\text{e}}-{\text{q}}_{\text{t}}\right)=\text{ln}{\text{q}}_{\text{e}}-{\text{k}}_{1}\text{t}$$ (6)

$$\text{t}/{\text{q}}_{\text{t}}=1/\left({\text{k}}_{2}{\text{q}}_{\text{e}}{}^{2}\right)-\text{t}/{\text{q}}_{\text{e}}$$ (7)

Where k_1_ represents the pseudo-first-order rate constant (min^−1^), k_2_ is the pseudo-second-order rate constant (g/mg·min^−1^) of adsorption, q_e_ and q_t_ are the amounts of Ni(II) adsorbed on MSBC (mg·g^-1^) at equilibrium and at time t, respectively.

The above parameters were obtained through curve fitting and presented in Table 3. The plotting of the graph ln (q_e_-q_t_) versus time (t) for the pseudo-first-order kinetic model did not yield good convergence (R^2^ 0.9435). In this regard, the correlation coefficient (R^2^ = 0.9995) of the pseudo-second-order model was remarkably higher than that of pseudo-first-order model. Furthermore, the equilibrium adsorption amount (q_e_) of the pseudo-second-order model (24.45 mg·g^-1^) was close to the experiment data (24.55 mg·g^-1^). Therefore, the kinetic analysis revealed the chemical adsorption of Ni onto MSBC was the dominating mechanism.

**Table 3:** Parameters of kinetic and isotherm models for Ni adsorption onto MSBC.

	Parameter 1	Parameter 2	R^2^
***Adsorption kinetics***			
Pseudo-first-order	k_1_ = 4.0×10^-3^min^-1^	q_e_ = 8.07 mg·g^-1^	0.9435
Pseudo-second-order	k_2_ = 6.31×10^−4^ g·mg·min^-1^	q_e_ = 24.45 mg·g^-1^	0.9995
***Adsorption isotherm***			
Langmuir	K_L_ = 3.56×10^−2^ L·mg^-1^	Q_m_ = 35.97 mg·g^-1^	0.9930
Freundlich	n = 5.7438	K_F_ = 10.50 mg·g^-1^	0.9821

#### Sorption isotherm

**Fig 5:**
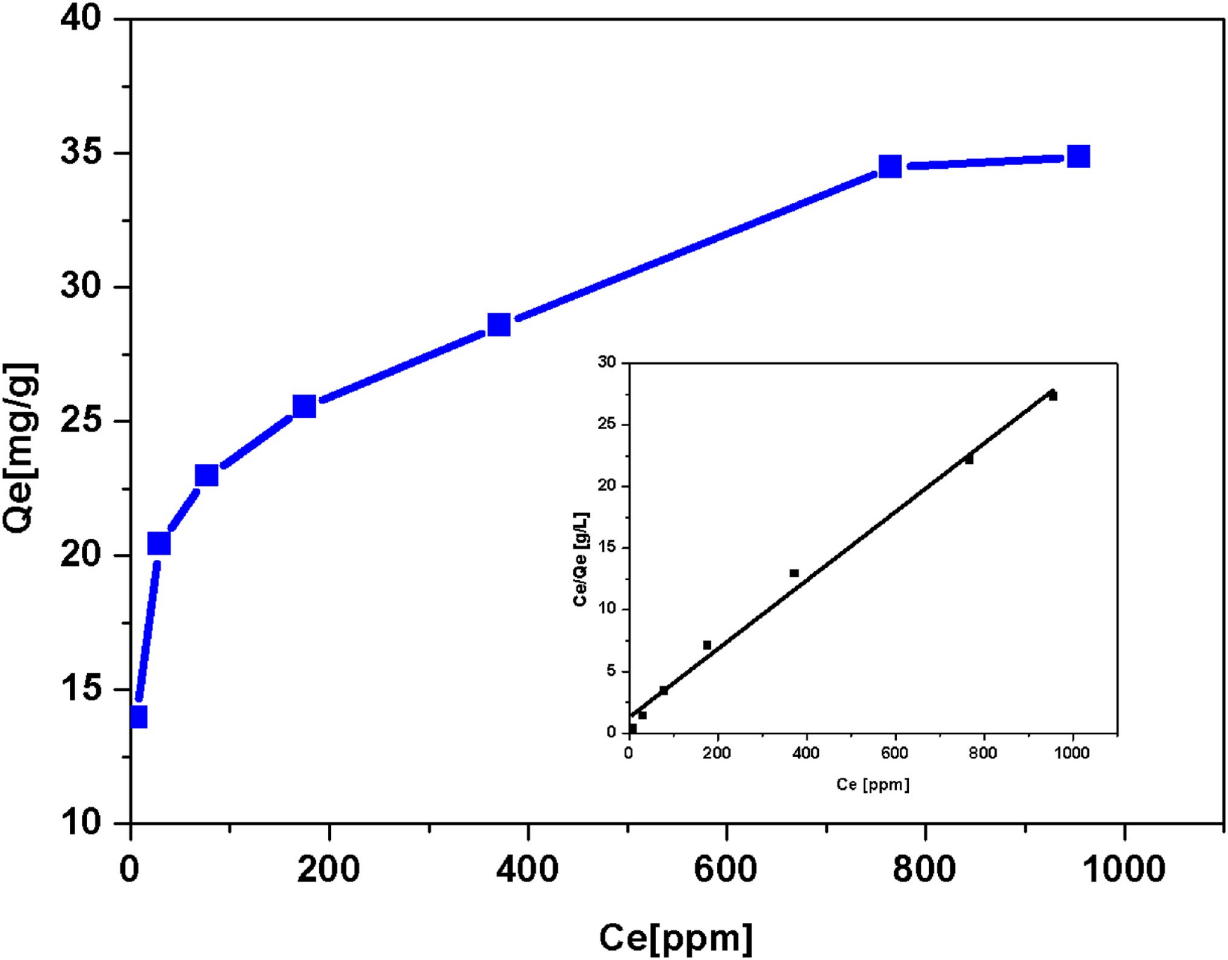
The isotherm of Ni(II) adsorption onto MSBC. Reaction conditions: Initial concentration of Ni(II) 20–1000 ppm at pH 7, 50 ml of solution, 50 mg of MSBC, 24 h, 25 °C.

As shown in Fig 5, the increased initial Ni(II) concentration (Ce) resulted in increased equilibrium adsorption capacity (Q_e_). Once the C_e_ reached above 800 ppm, no further increase was observed for Q_e_. The Langmuir and Freundlich models, frequently used in various adsorption studies [11, 39], were employed to simulate the isotherm of Ni(II) on MSBC (Table 3). Obviously, the experimental data was well fitted by the Langmuir model with an R^2^ value of 0.9930, while that of the Freundlich model was 0.9821. Actually, the adsorption path of the Langmuir model was believed to be monolayer sorption on a homogeneous sorption surface [40]. Nevertheless, the Freundlich model can be regard as non-ideal sorption on a heterogeneous surface as well as multilayer sorption [40]. As mentioned above, the R^2^ value of the Langmuir model was slightly higher than that of the Freundlich model. The calculated values of Ni(II) adsorption capacity were 35.97 mg·g^-1^ (Q_m_) and 10.50 mg·g^-1^ (K_L_), respectively for the Langmuir and Freundlich models. Comparing to the adsorption capacity of MSBC (Table 2), Q_m_ was much closer to 35.50 mg·g^-1^ than K_L_. This suggested that monolayer sorption on a homogeneous sorption surface could be dominant in our experiments.

### Recycle and desorption performance

**Fig 6:**
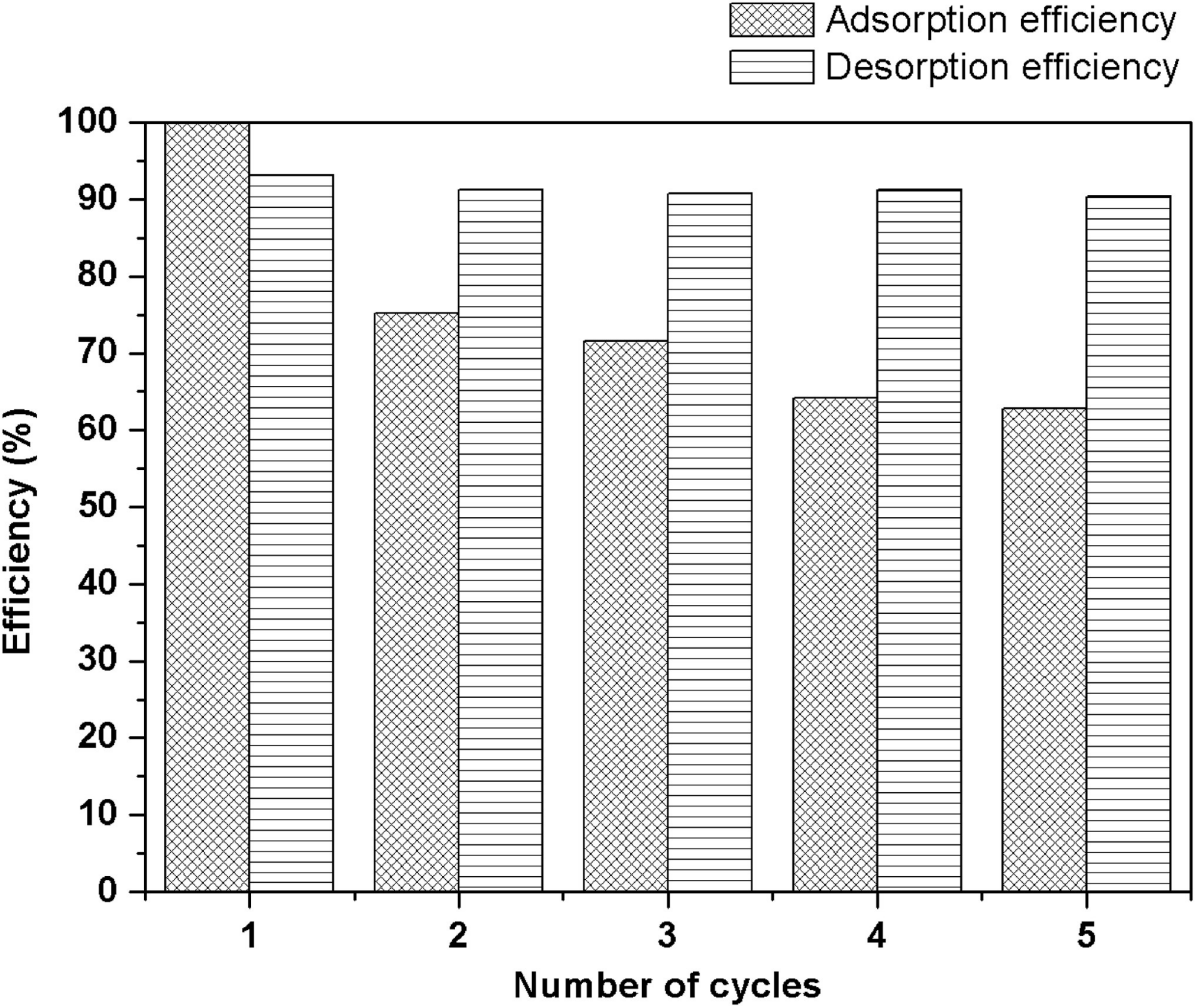
Ni(II) adsorption or desorption efficiencies of MSBC at five regeneration cycles.

The regeneration capability of the biochar is vital for further application from an economic point of view. Desorption attempts could be applied to recycle the metal ions and regenerate the saturated adsorbents. Thermal activation and acid solution were reported as regeneration methods based on previous literatures [40, 44]. High energy requirement and adsorbent loss were noticed in thermal activation attempts of the saturated adsorbent [44]. Acid desorption may cause negative impact of the adsorption abilities and structure of the regenerated biochar [40]. So far Na_2_EDTA was found effective in the desorption of Pb(II) from the saturated biochar with less negative effects[14]. In this study, 0.1 M Na_2_EDTA was employed as a desorption agent. The adsorption and desorption efficiencies of MSBC for five cycles are shown in Fig 6. It was observed that the removal efficiency remained 60% and the desorption efficiency still achieved around 90% in the fifth cycle. The remarkable performance of MSBC demonstrated excellent regeneration potential, which presented the prospect of its expanding lifespan. The contents of typical heavy metals in SBC and MSBC were also investigated (S1 Table). Compared with the threshold values for sewage sludge used in forestland, all the heavy metals contents in the SBC and MSBC were lower than those in the Chinese standard [45]. In addition, the exchangeable and acid-soluble fraction and the reducible fraction of heavy metals could be decreased via pyrolysis [46]. Overall, the MSBC adsorbent produced in this study is safe for the natural environment.

### Adsorption mechanism

The mechanism of Ni(II) adsorption on adsorbents was believed to be the synergistic effect of physical and chemical adsorptions [7]. The high surface areas and pore structure of MSBC were observed based on BET and SEM analysis. Besides, batch experiments at various pH values indicated that the electrostatic attraction occurred in the adsorption process. However, the dominant role was proved to be chemical adsorption rather than physical adsorption based on our kinetic study. Similar results were reported in other Ni(II) adsorption studies [47]. Furthermore, the adsorption isotherm analysis revealed monolayer sorption on a homogeneous sorption surface could be dominant in the experiments. The initial rapid adsorption phenomenon could be attribute to electrostatic attraction and ion exchange, and chemical adsorption acted as a crucial role in the following step [14]. The chemical reactions of heavy metal adsorption were generally defined as inner-sphere complexation and co-precipitation [47]. The solution after Ni(II) adsorption was detected by ICP-AES and K^+^, Mg^2+^ and Ca^2+^ were found after the adsorption treatment, convincingly demonstrating the ion exchange between Ni(II) and aforementioned metal ions. Comparison analysis was achieved between the FTIR spectra of MSBC before and after Ni(II) adsorption (S4 Fig). Significant shifts were found from 3420 cm^-1^ to 3397 cm^-1^, 1635 cm^-1^ to 1631 cm^-1^, 1094 cm^-1^ to 1077 cm^-1^, 1033 cm^-1^ to 1030 cm^-1^, 796 cm^-1^ to 797 cm^-1^, 780 cm^-1^ to 779 cm^-1^, 693 cm^-1^ to 694 cm^-1^, 548 cm^-1^ to 550 cm^-1^, and 472 cm^-1^ to 475 cm^-1^. The band change from 548 cm^-1^ to 550 cm^-1^ could be attributed to the O-H from α-Fe_2_O_3_, due to about 4–10 iron-bonded hydroxyl groups per nm^2^ on the surface of α-Fe_2_O_3_ [25]. According to the wavelength variation of O-H, Ni(II) was probably bonded with free hydroxyl (O-H) functional groups by way of inner-sphere complexation like other heavy metal ions [14]. Meanwhile, co-precipitation reaction between Ni(II) and PO_4_^3−^ might occur, which was suggested by the peak variation from 1094 cm^-1^ to 1077 cm^-1^ (P-O). In addition, the peak at 602 cm^-1^ disappeared after adsorption, which suggested the involvement of Fe-OH. The interaction between α-FeOOH and Cu^2+^ was found possibly via the formation of Fe-O-Cu complexes [23]. The formation of Fe-O-Ni complexes occurred probably during the adsorption process in this study. In short, inner-sphere complexation and co-precipitation could be confirmed based on the above FTIR analysis. Furthermore, the comparison of XRD patterns between MSBC before and after Ni(II) adsorption was conducted to understand the Ni formations via the crystalline structure analysis. The XRD patterns of Ni_3_(PO_4_)_2_ (PDF#38–1473) and Ni(OH)_2_ (PDF#38–0715) co-precipitations were identified by XRD analysis with the typical 2 theta of 24.12°, 30.03°, 38.20°, 53.17°, 63.89° and 22.61°, 45.83°, 59.93°, 61.32°, respectively (S5 Fig). The formation of Ni_3_(PO_4_)_2_ and Ni(OH)_2_ may be due to reactions (8) and (9). The outcome of XRD analysis was consistent with that of the FTIR spectra. The high content of inorganic phosphorus (S2 Table) in MSBC also supported the formation of Ni_3_(PO_4_)_2_.

$${\text{Ni}}^{2+}+2{\text{OH}}^{-}\to \text{Ni}{\left(\text{OH}\right)}_{2}↓$$ (8)

$$3{\text{Ni}}^{2+}+2{\left({\text{PO}}_{4}\right)}^{3+}\to {\text{Ni}}_{3}{\left({\text{PO}}_{4}\right)}_{2}↓$$ (9)

In conclusion, Ni adsorption was mainly attributed to electrostatic attraction, ion exchange, inner-sphere complexation and co-precipitation. Fast adsorption in the first several minutes was due to electrostatic attraction and ion exchange. And then the crucial mechanisms were inner-sphere complexation and co-precipitation.

## Conclusions

Modified sewage sludge biochar (MSBC) was technically produced by distributing both α-Fe_2_O_3_ and α-FeOOH particles onto the surface of SBC. The maximum Ni adsorption capacity of MSBC reached 35.50 mg·g^-1^. The adsorption process could be fitted by the pseudo-second-order model and adsorption isotherm was well described by the Langmuir model. The initial rapid adsorption phenomenon could be attributed to electrostatic attraction and ion exchange, and then inner-sphere complexation and co-precipitation acted as a crucial role in the following step. Although the mechanism of Ni(II) adsorption was believed to be the synergistic effect of physical and chemical adsorptions, the dominant role was proved to be chemical adsorption. The results in this study indicate that MSBC could act as an excellent adsorbent for Ni removal.

## Supporting information

S1 Table Total concentrations of heavy metals in samples and their threshold values for the disposal standards of China. (DOC)

S2 Table The inorganic phosphorus contents of SBC and MSBC. (DOC)

S1 Fig XRD patterns of SBC, MSBC before nickel adsorption. (TIF)

S2 Fig SEM images of (a) SSBC (X2000), (b) SSBC (X5000), (c) MSSBC (X2000) and (d) MSSBC (X5000). (TIF)

S3 Fig FTIR spectra of SBC and MSBC before nickel adsorption. (TIF)

S4 Fig FTIR spectra of MSBC before and after nickel adsorption. (TIF)

S5 Fig XRD spectra of MSBC after nickel adsorption. (TIF)

S1 Data Raw Data. (ZIP)
