# Supplementary material for: Highly efficient nickel (II) removal by sewage sludge biochar supported α-Fe2O3 and α-FeOOH: Sorption characteristics and mechanisms
Source: PLoS One. 2019 Jun 12;14(6):e0218114. doi: 10.1371/journal.pone.0218114 (PMC6561682; doi:10.1371/journal.pone.0218114)
Supplement: S1 Table — (DOC) [file pone.0218114.s001.doc]

**S1 Table. Total concentrations of heavy metals in samples and their threshold values for the disposal standards of China**

| Sample | Heavy metals (mg/kg) | | | | |  |
| --- | --- | --- | --- | --- | --- | --- |
|  | Pb | Cd | Cr | Hg | As | Ni |
| Dry sludge | 42.7 | 13.2 | 117.0 | 4.0 | 20.4 | 23.2 |
| SBC | 20.4 | 17.4 | 209.0 | 5.2 | 29.6 | 43.6 |
| MSBC | 36.7 | 15.5 | 188.8 | 2.6 | 18.4 | 29.5 |
| Threshold values ^a^ | 1000 | 20 | 1000 | 15 | 75 | 200 |

^a^ According to the disposal of sludge from municipal wastewater treatment plant-Quality of sludge used in forestland (CJ/T 362-2011).
