# Supplementary material for: Highly efficient nickel (II) removal by sewage sludge biochar supported α-Fe2O3 and α-FeOOH: Sorption characteristics and mechanisms
Source: PLoS One. 2019 Jun 12;14(6):e0218114. doi: 10.1371/journal.pone.0218114 (PMC6561682; doi:10.1371/journal.pone.0218114)
Supplement: S2 Table — (DOC) [file pone.0218114.s002.doc]

**S2 Table. The inorganic phosphorus contents of SBC and MSBC**

| Biochar | SBC | MSBC |
| --- | --- | --- |
| P content (% weight) | 1.02 | 0.89 |
