# Supplementary figures and images for: Highly efficient nickel (II) removal by sewage sludge biochar supported α-Fe2O3 and α-FeOOH: Sorption characteristics and mechanisms
Source: PLoS One. 2019 Jun 12;14(6):e0218114. doi: 10.1371/journal.pone.0218114 (PMC6561682; doi:10.1371/journal.pone.0218114)

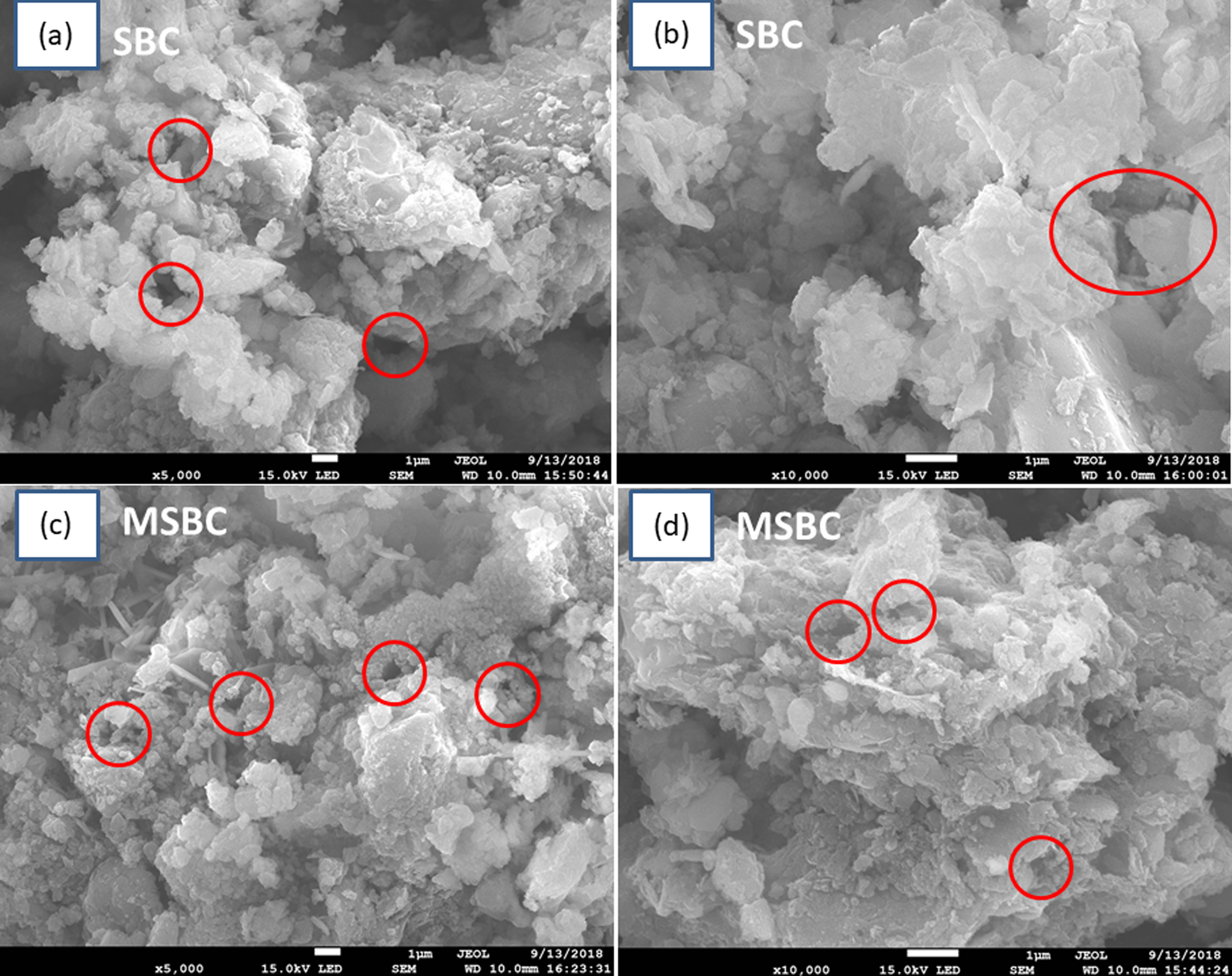

Supplement: S2 Fig — (TIF) [file pone.0218114.s004.tif]

| 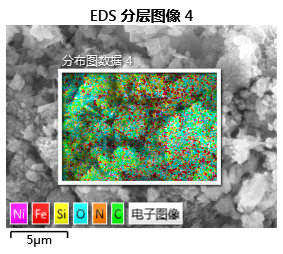 | 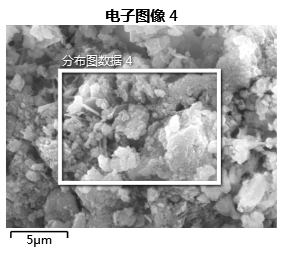 |
| --- | --- |


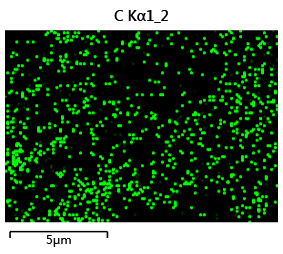

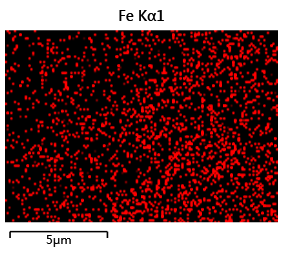

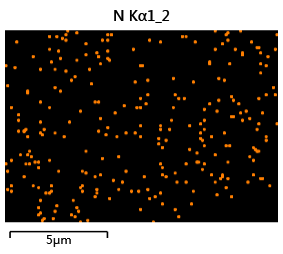

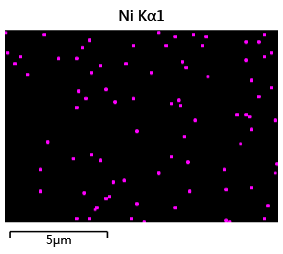

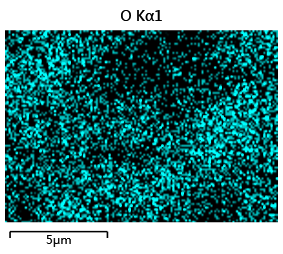

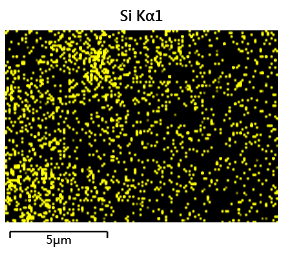

Supplement: S1 Data — (ZIP) [file pone.0218114.s008.zip › Raw data/Characteristics/EDS/Data/reports/1_2018-09-13_16-30-21.docx]

| 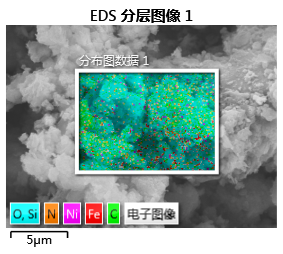 | 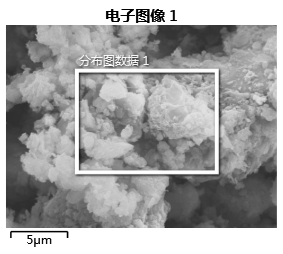 |
| --- | --- |


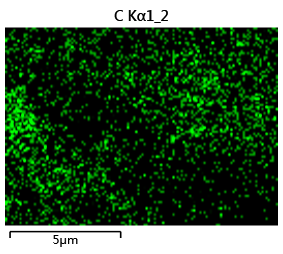

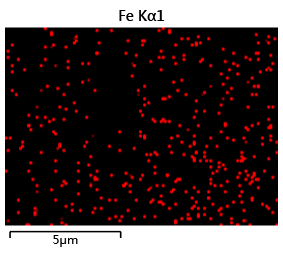

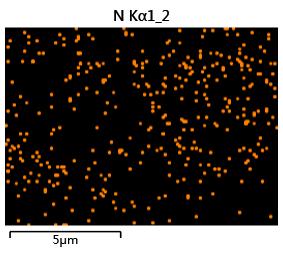

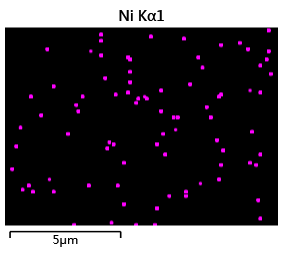

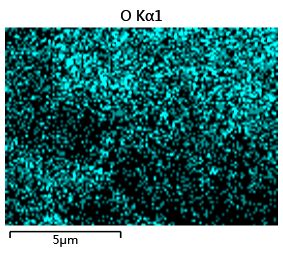

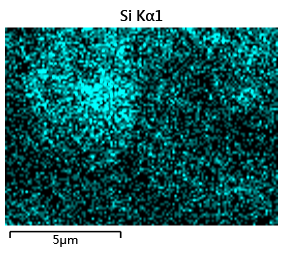

Supplement: S1 Data — (ZIP) [file pone.0218114.s008.zip › Raw data/Characteristics/EDS/Data/reports/2_2018-09-13_16-00-07.docx]

| 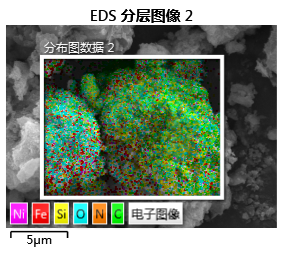 | 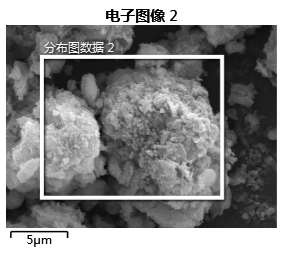 |
| --- | --- |


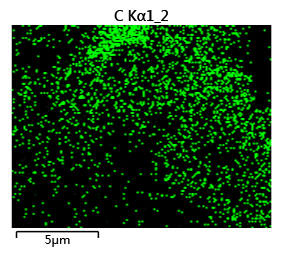

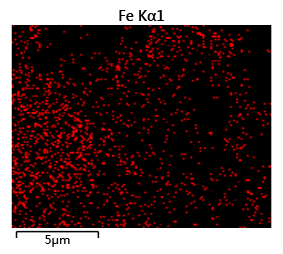

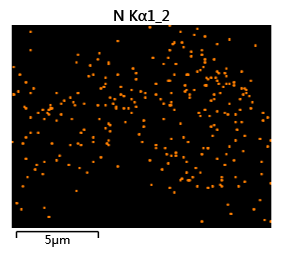

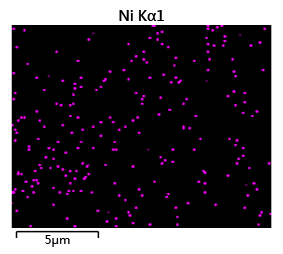

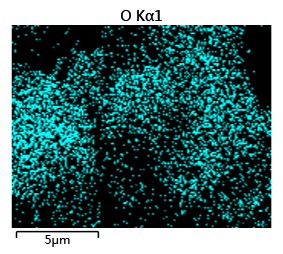

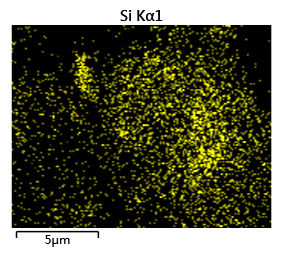

Supplement: S1 Data — (ZIP) [file pone.0218114.s008.zip › Raw data/Characteristics/EDS/Data/reports/3_2018-09-13_16-13-11.docx]

| 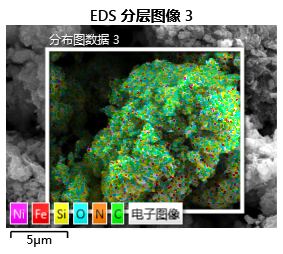 | 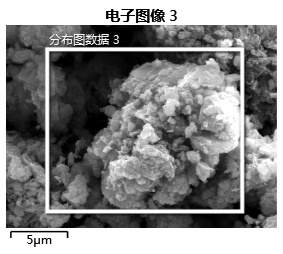 |
| --- | --- |


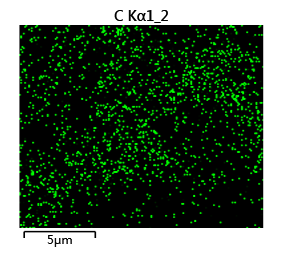

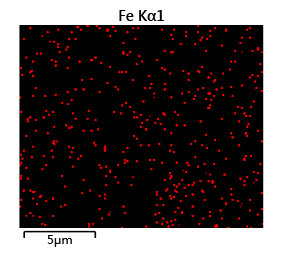

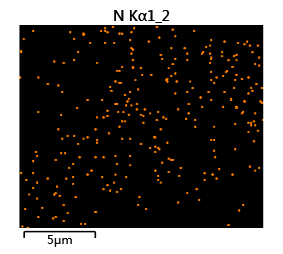

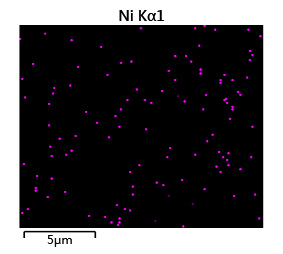

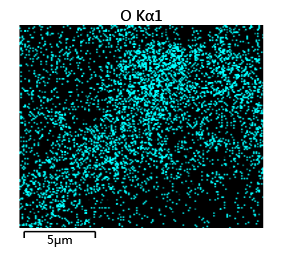

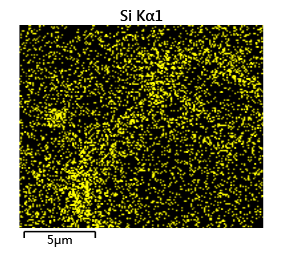

Supplement: S1 Data — (ZIP) [file pone.0218114.s008.zip › Raw data/Characteristics/EDS/Data/reports/4_2018-09-13_16-21-49.docx]

## Slide 1
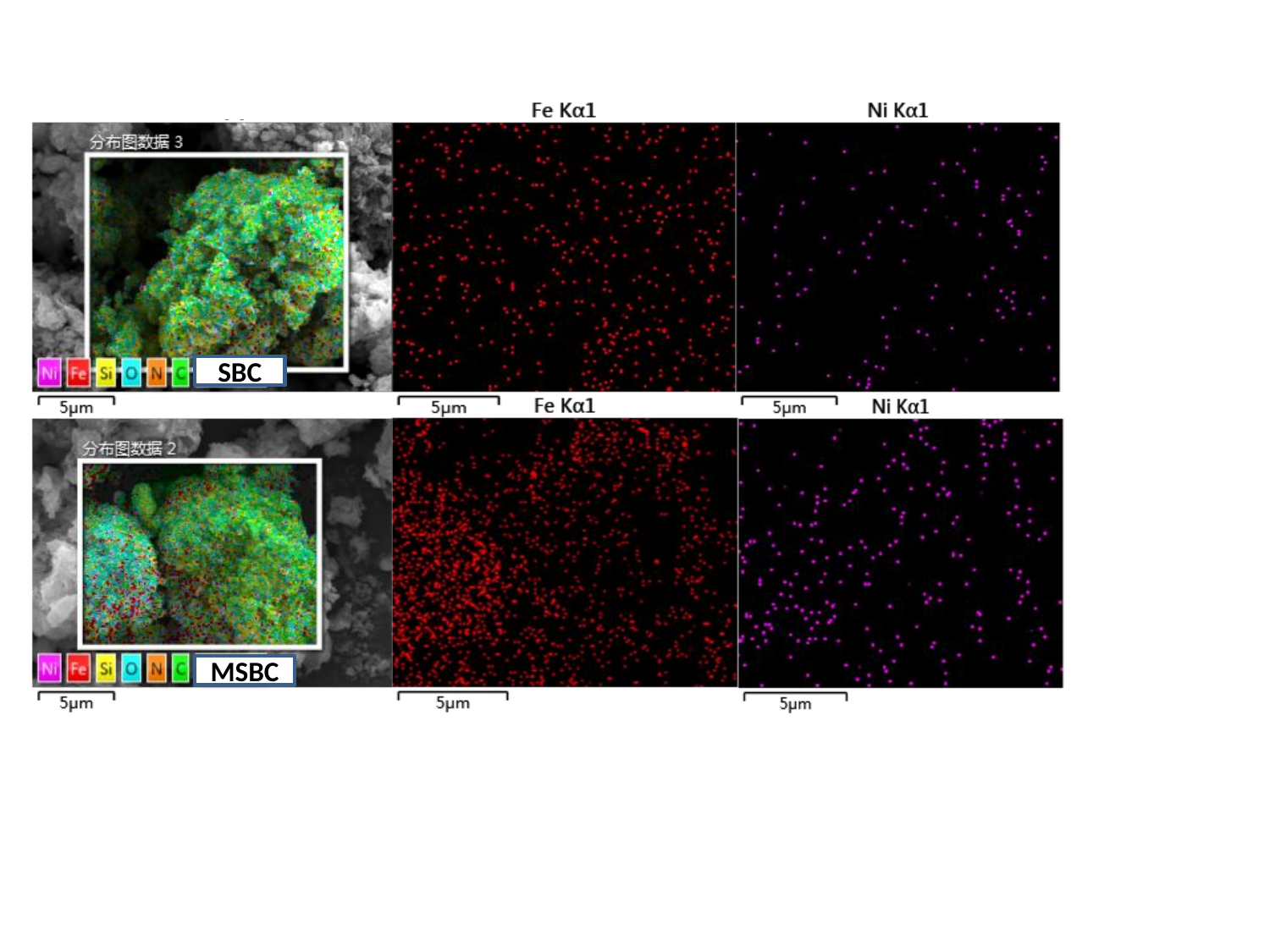

SBC
MSBC

Supplement: S1 Data — (ZIP) [file pone.0218114.s008.zip › Raw data/Characteristics/Figures/EDS/EDS.pptx]

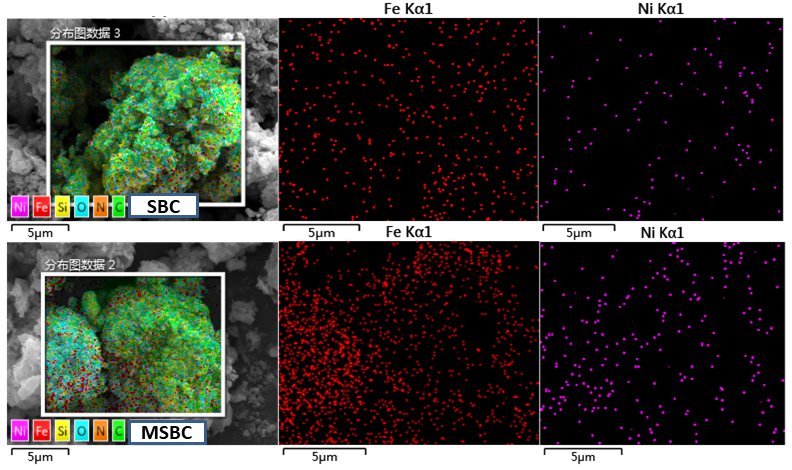

Supplement: S1 Data — (ZIP) [file pone.0218114.s008.zip › Raw data/Characteristics/Figures/EDS/EDS.tif]

## Slide 1
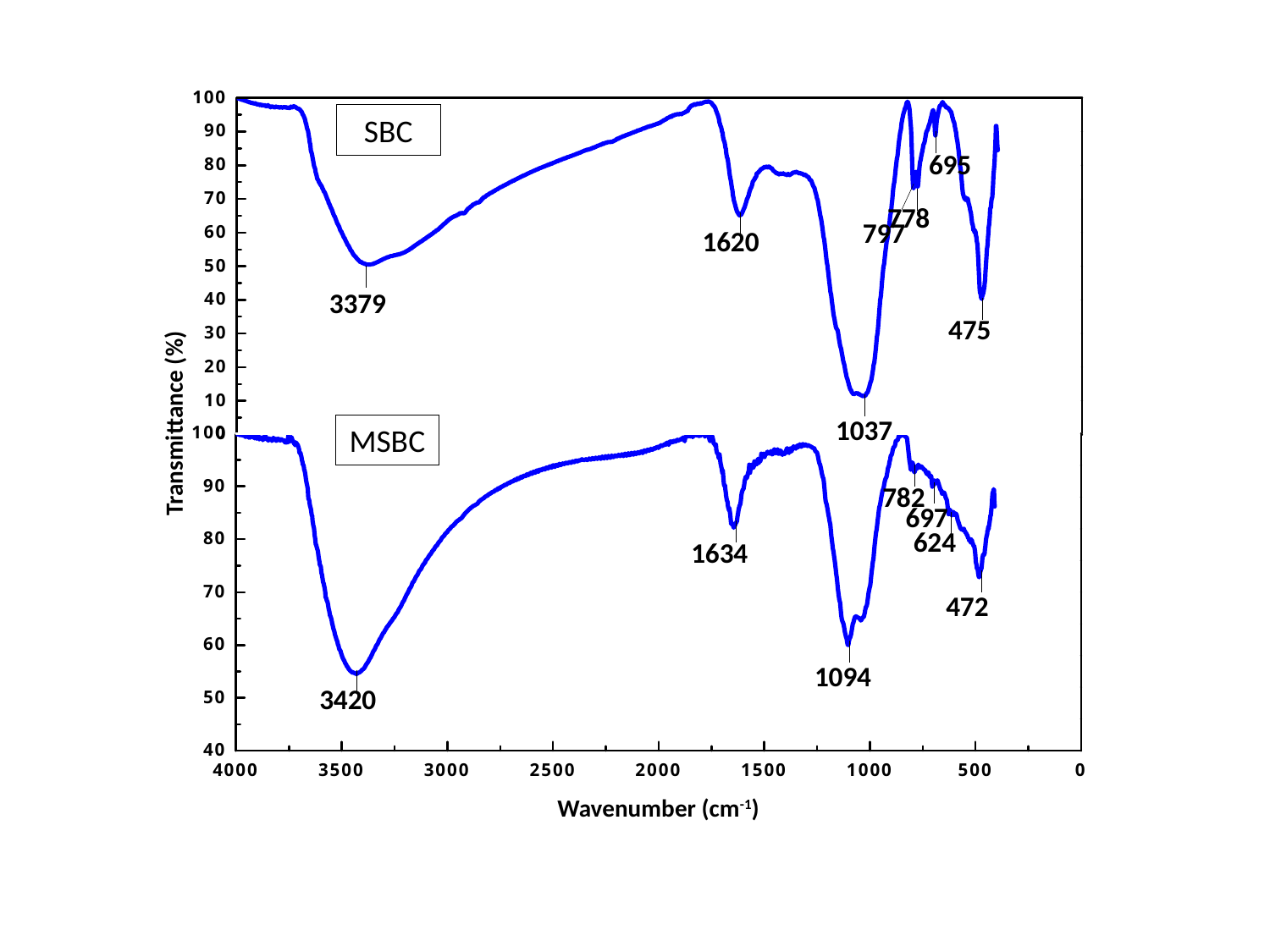

SBC
695
778
797
1620
3379
475
Transmittance (%)
1037
MSBC
782
697
624
1634
472
1094
3420
Wavenumber (cm-1)

Supplement: S1 Data — (ZIP) [file pone.0218114.s008.zip › Raw data/Characteristics/Figures/FTIR/FTIR-1.pptx]

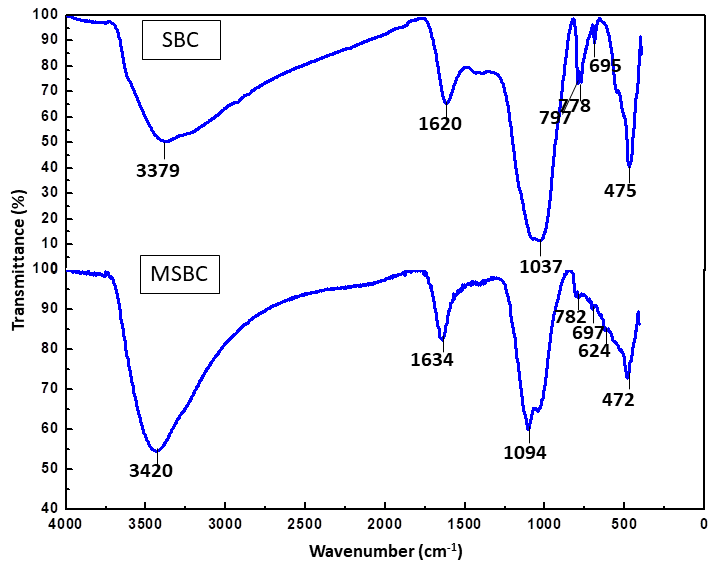

Supplement: S1 Data — (ZIP) [file pone.0218114.s008.zip › Raw data/Characteristics/Figures/FTIR/FTIR-1.tif]

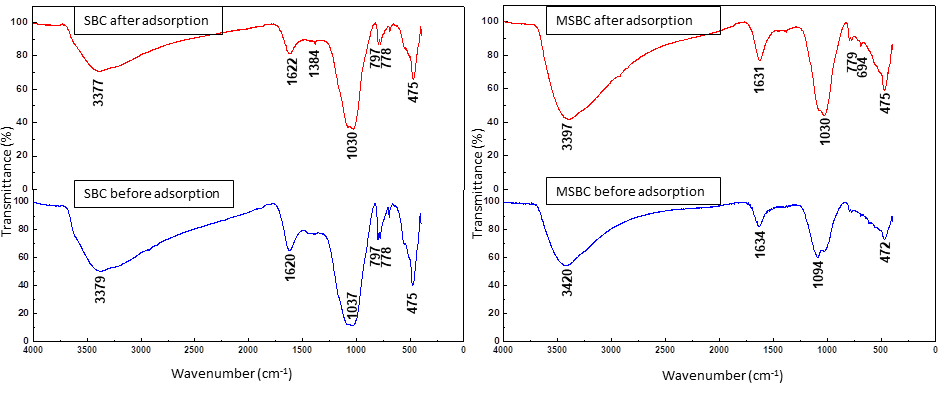

Supplement: S1 Data — (ZIP) [file pone.0218114.s008.zip › Raw data/Characteristics/Figures/FTIR/FTIR-2.tif]

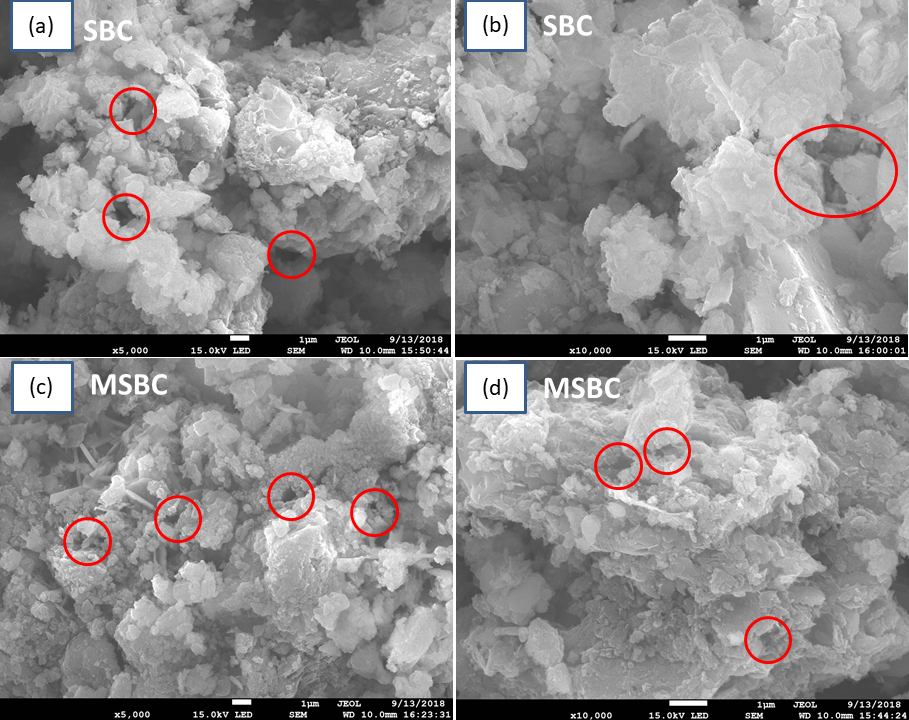

Supplement: S1 Data — (ZIP) [file pone.0218114.s008.zip › Raw data/Characteristics/Figures/SEM/SEM.TIF]

## Slide 1
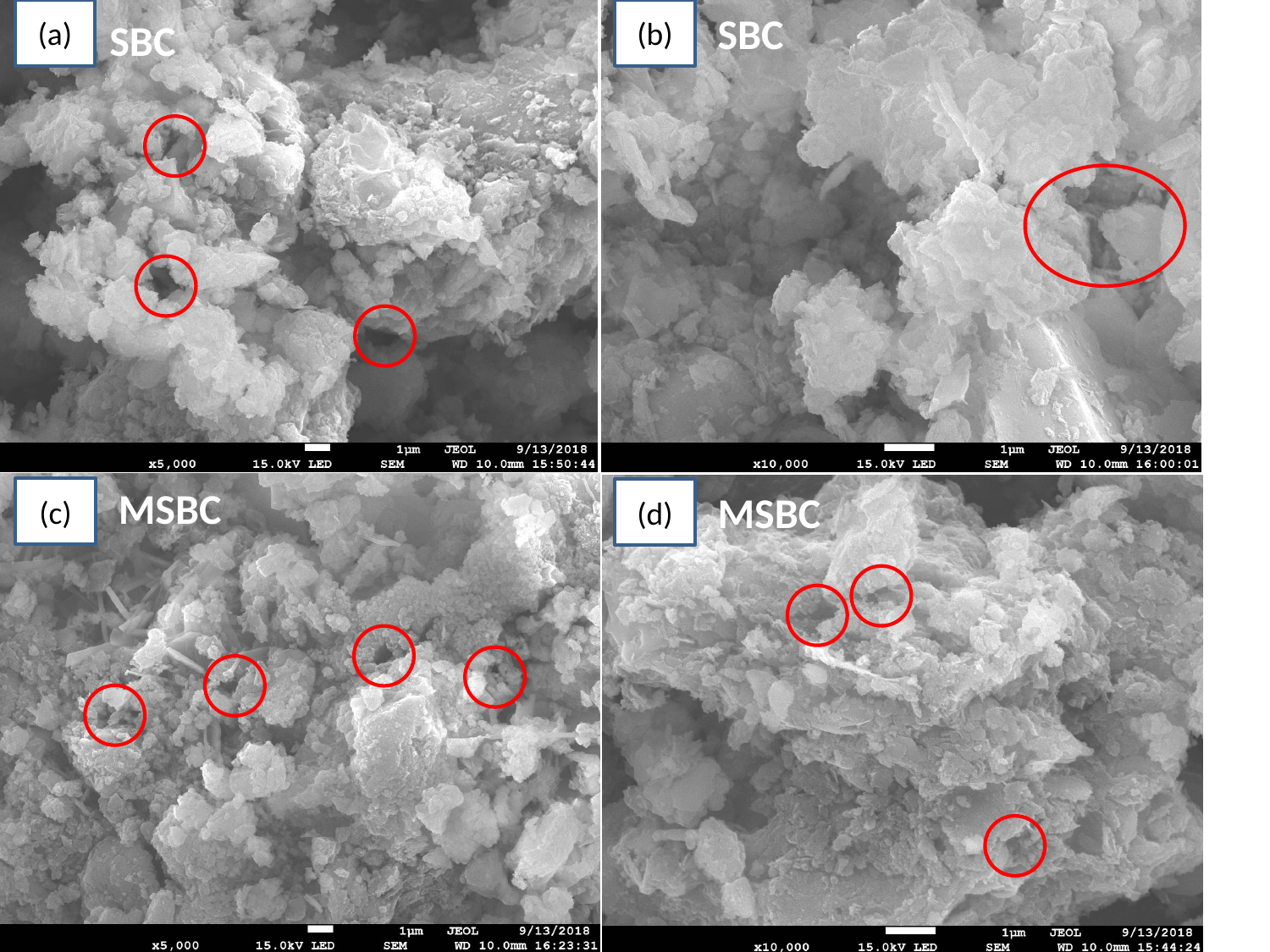

(a)
(b)
SBC
SBC
#
MSBC
(c)
(d)
MSBC

Supplement: S1 Data — (ZIP) [file pone.0218114.s008.zip › Raw data/Characteristics/Figures/SEM/SEM.pptx]

## Slide 1
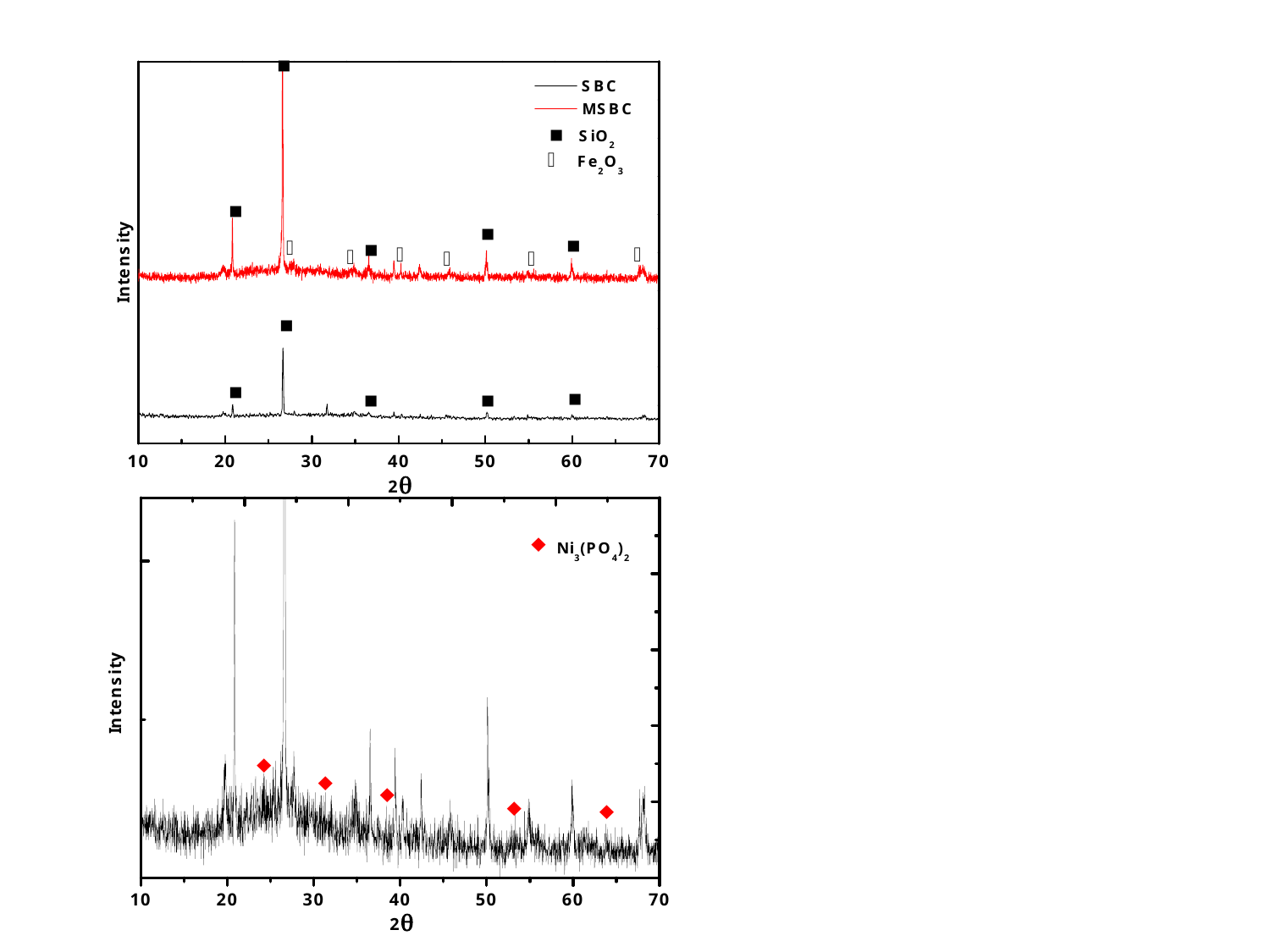

Supplement: S1 Data — (ZIP) [file pone.0218114.s008.zip › Raw data/Characteristics/Figures/XRD/XRD.pptx]

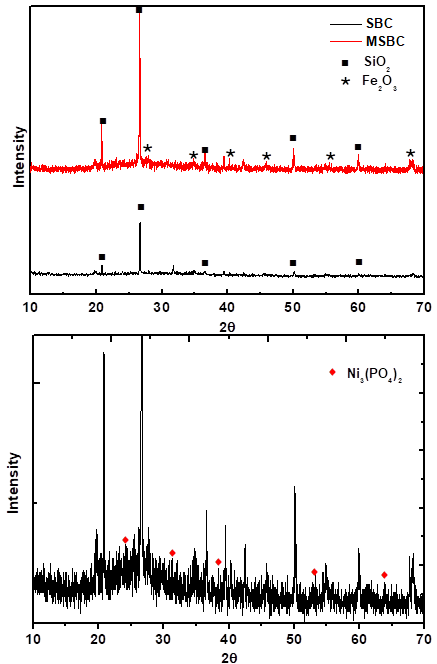

Supplement: S1 Data — (ZIP) [file pone.0218114.s008.zip › Raw data/Characteristics/Figures/XRD/XRD.tif]

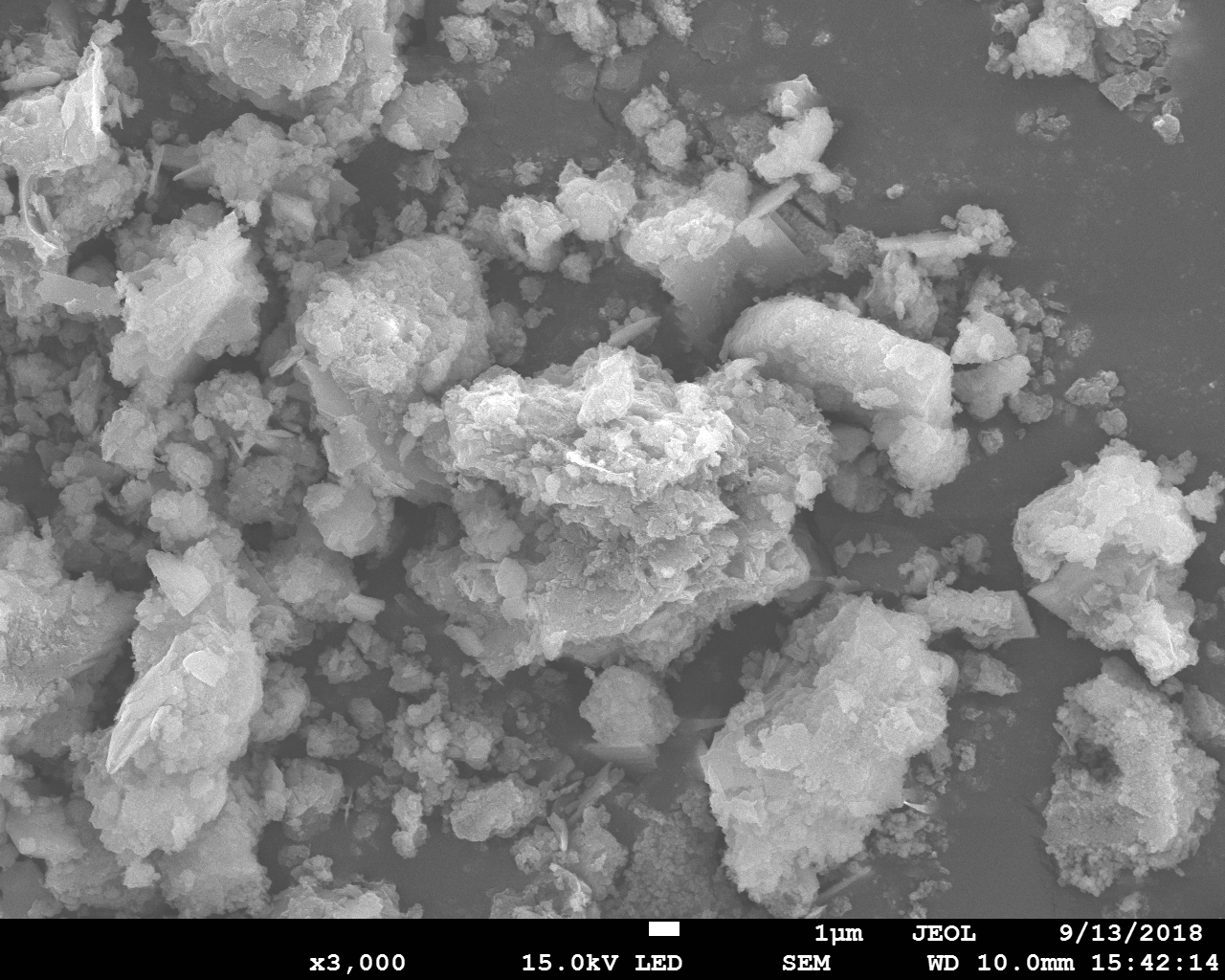

Supplement: S1 Data — (ZIP) [file pone.0218114.s008.zip › Raw data/Characteristics/SEM/+1/1-1-1.bmp]

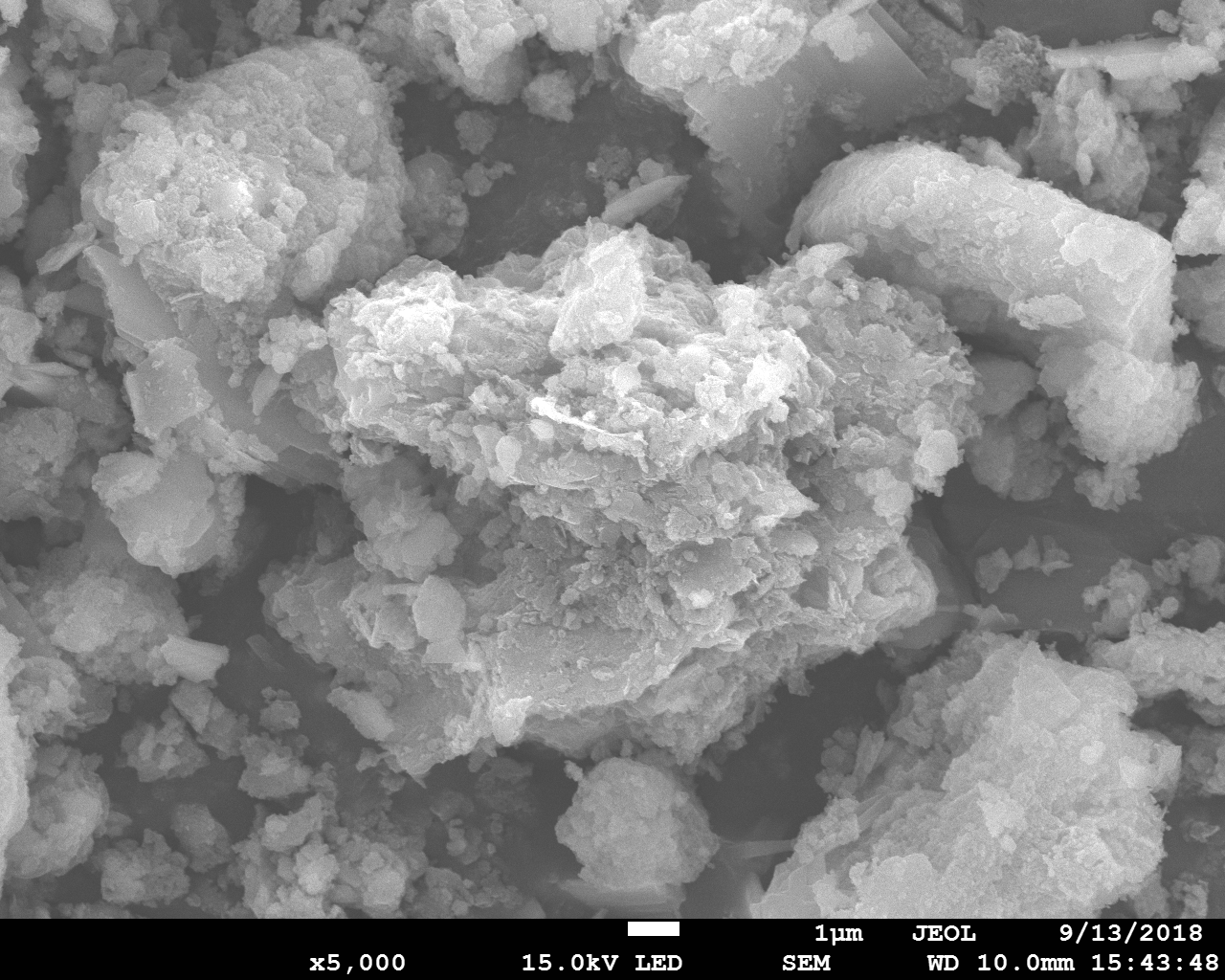

Supplement: S1 Data — (ZIP) [file pone.0218114.s008.zip › Raw data/Characteristics/SEM/+1/1-1-2.bmp]

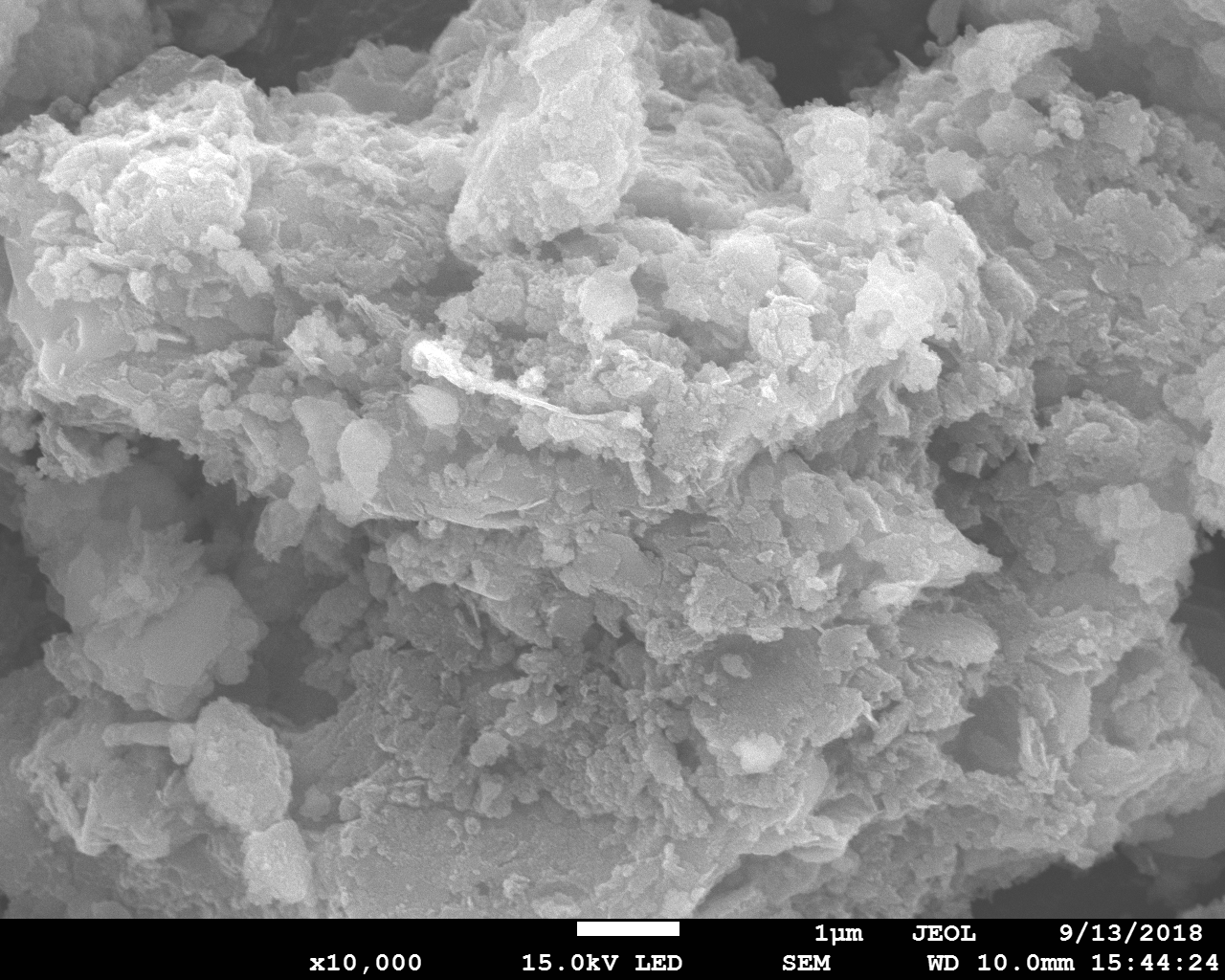

Supplement: S1 Data — (ZIP) [file pone.0218114.s008.zip › Raw data/Characteristics/SEM/+1/1-1-3.bmp]

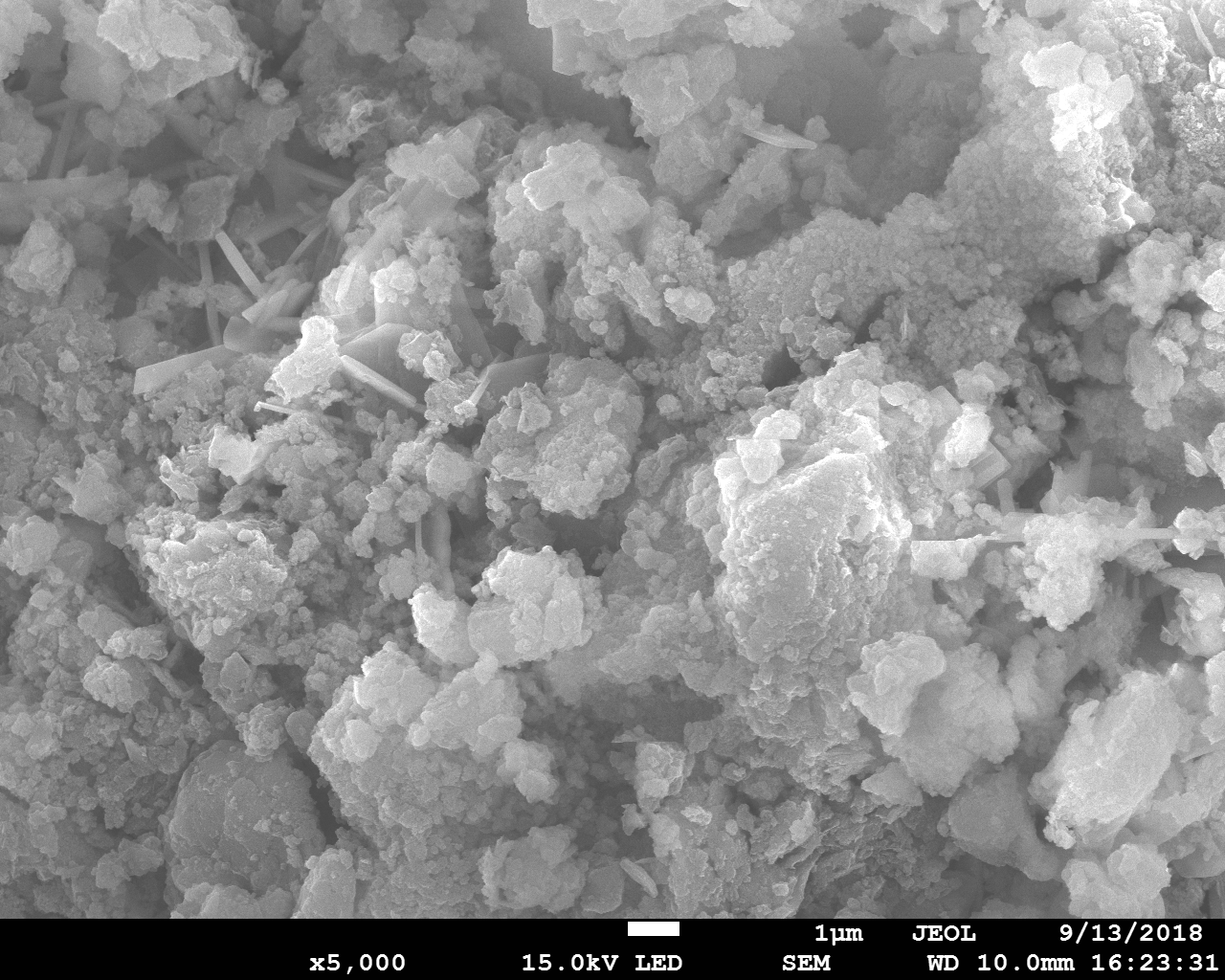

Supplement: S1 Data — (ZIP) [file pone.0218114.s008.zip › Raw data/Characteristics/SEM/+1/1-1-4.bmp]

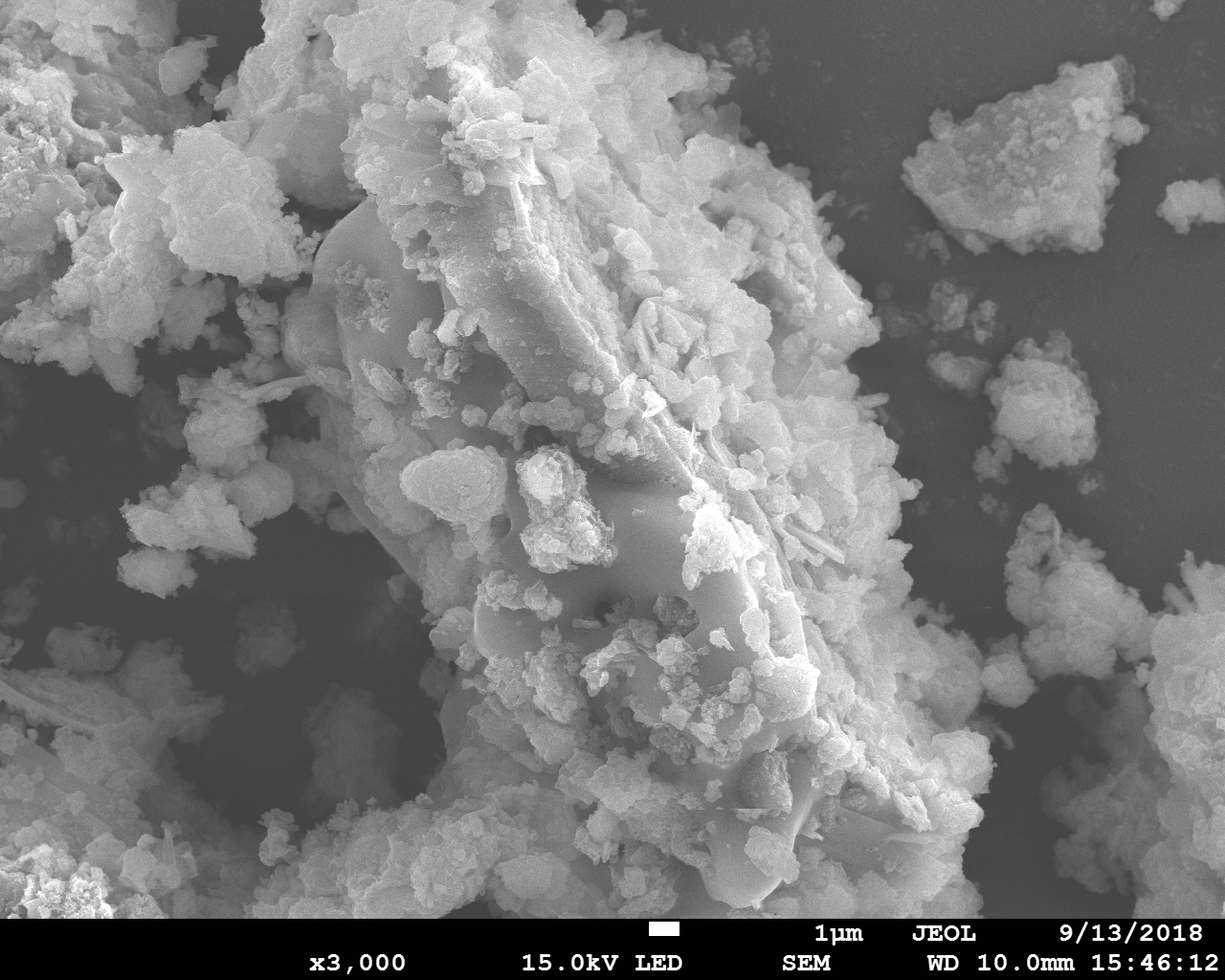

Supplement: S1 Data — (ZIP) [file pone.0218114.s008.zip › Raw data/Characteristics/SEM/+1/1-2-1.bmp]

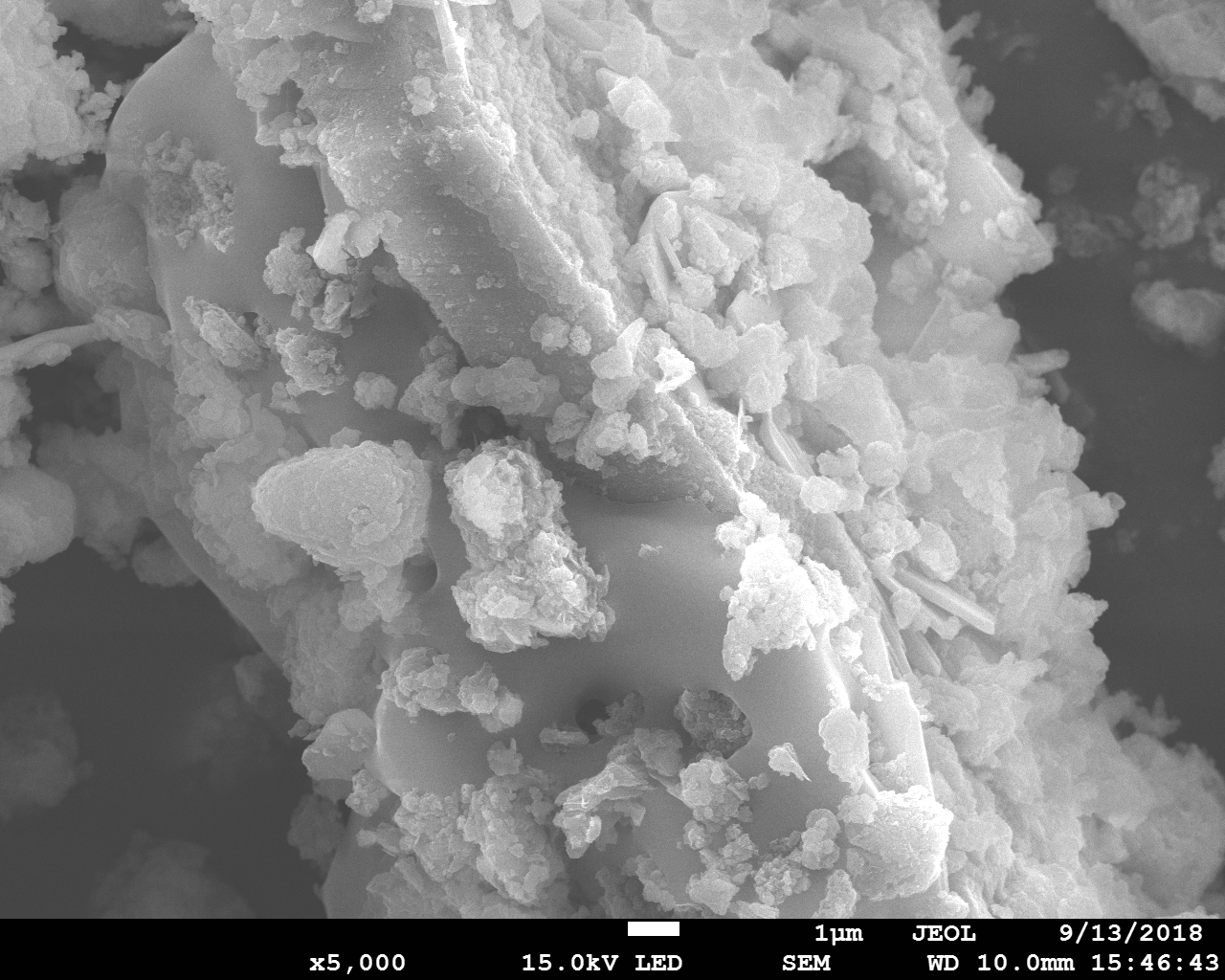

Supplement: S1 Data — (ZIP) [file pone.0218114.s008.zip › Raw data/Characteristics/SEM/+1/1-2-2.bmp]

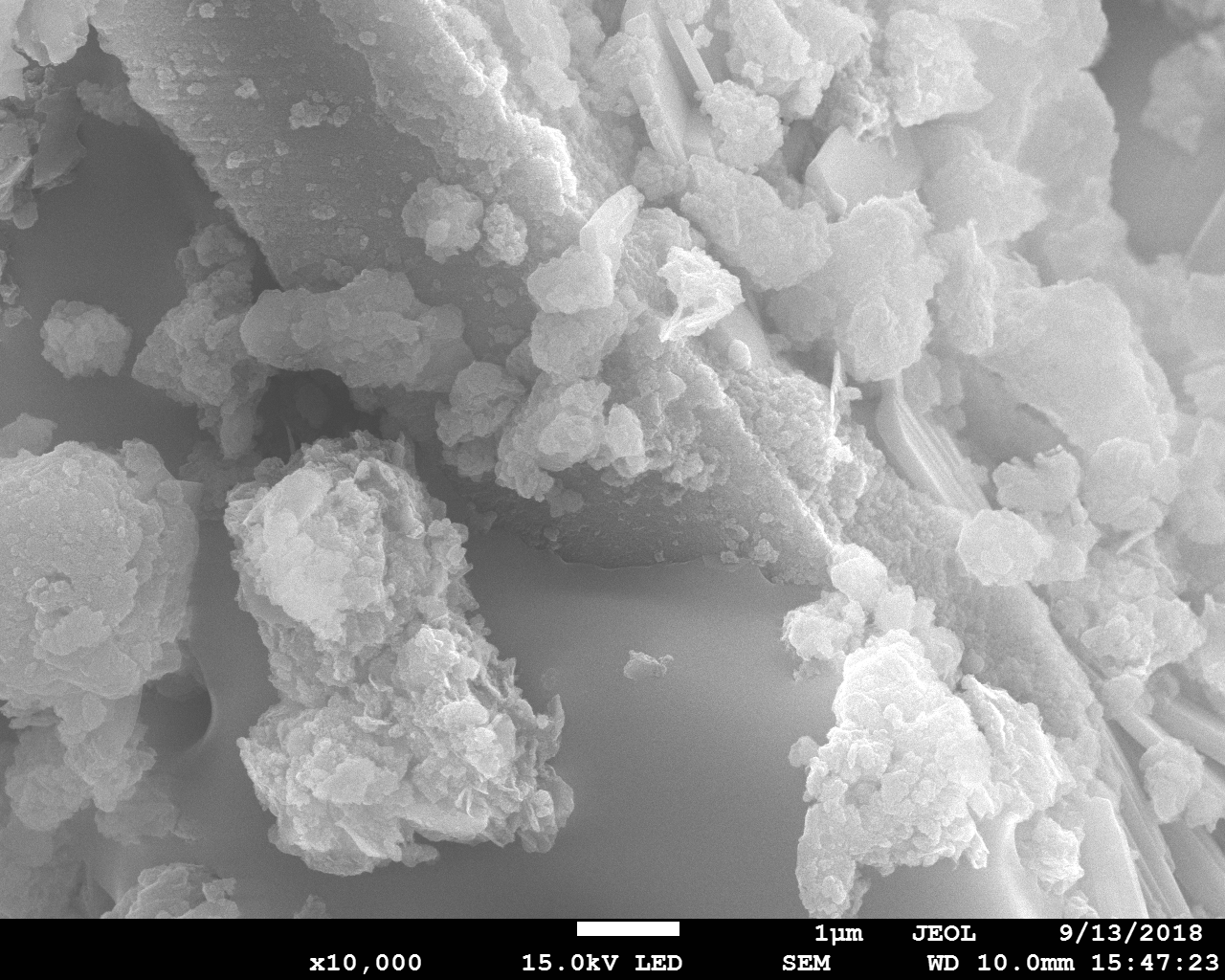

Supplement: S1 Data — (ZIP) [file pone.0218114.s008.zip › Raw data/Characteristics/SEM/+1/1-2-3.bmp]

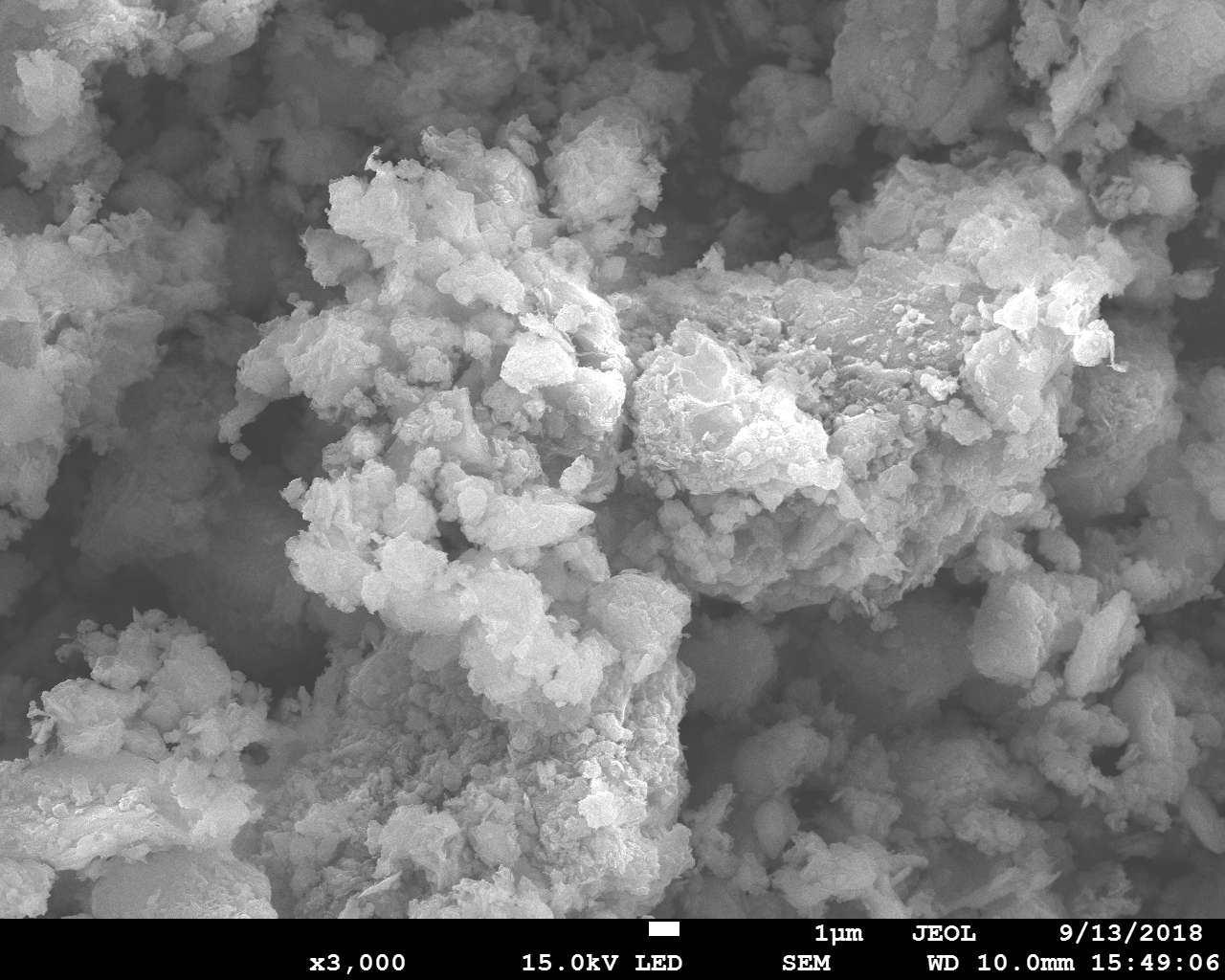

Supplement: S1 Data — (ZIP) [file pone.0218114.s008.zip › Raw data/Characteristics/SEM/+1/2-1-1.bmp]

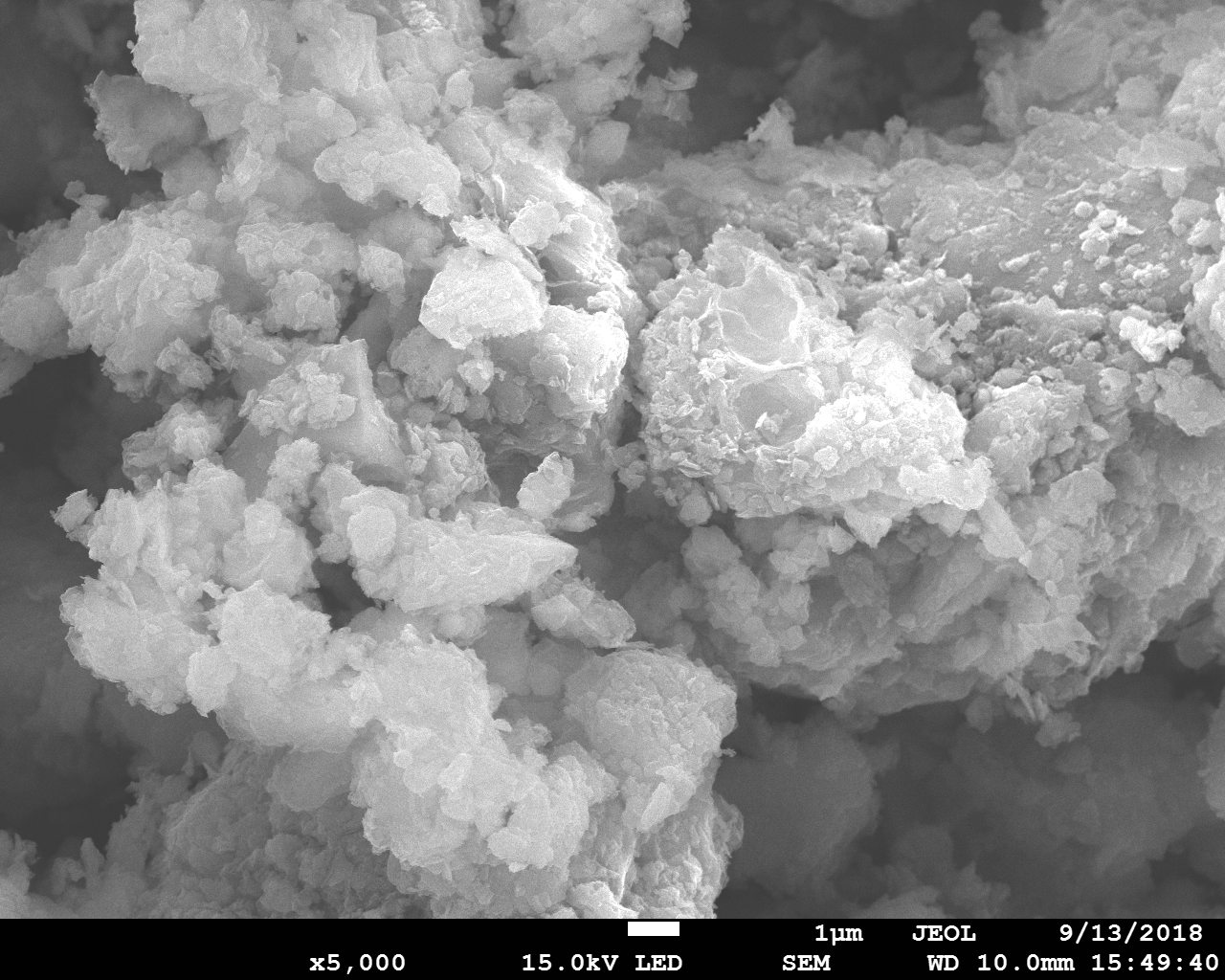

Supplement: S1 Data — (ZIP) [file pone.0218114.s008.zip › Raw data/Characteristics/SEM/+1/2-1-2.bmp]

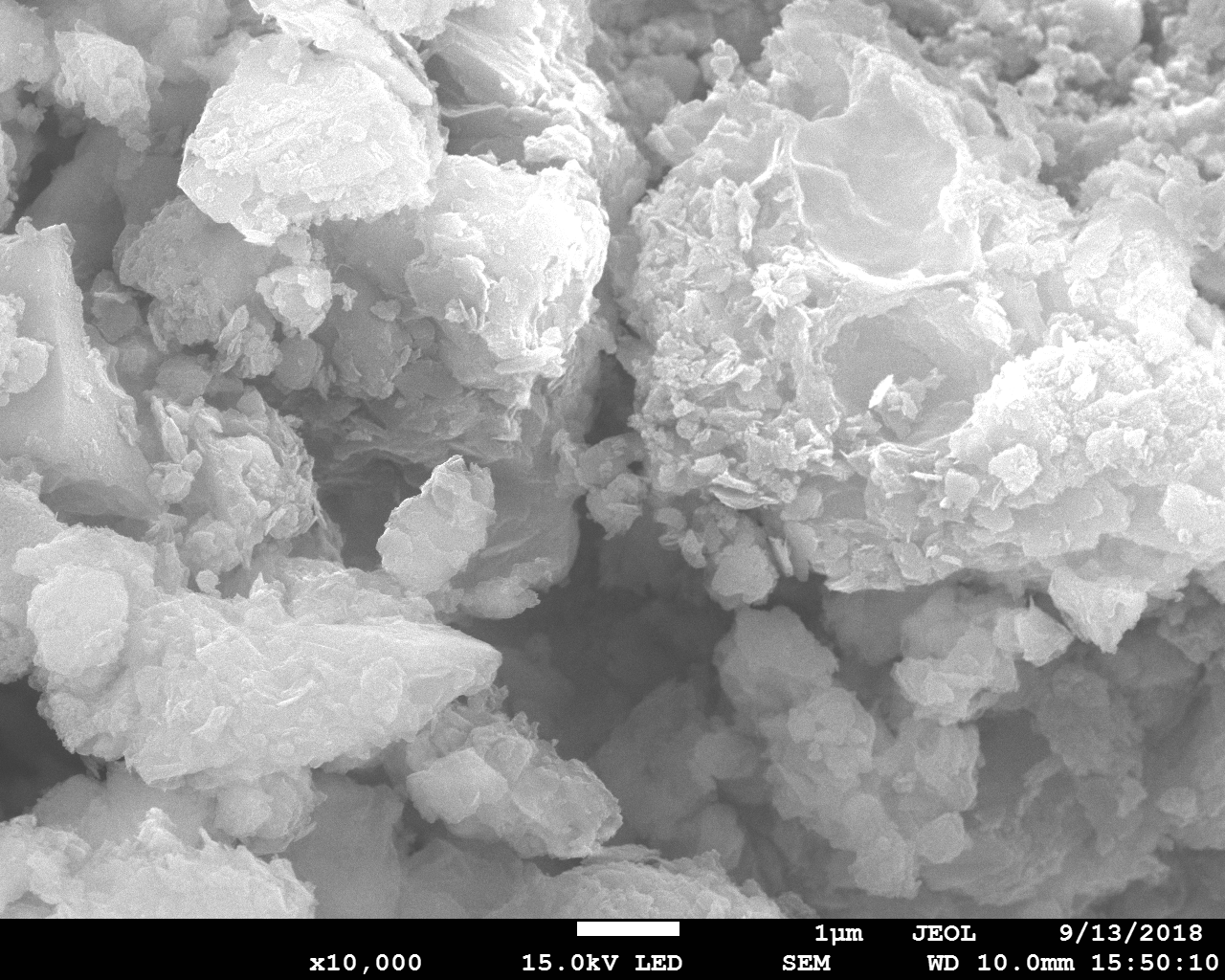

Supplement: S1 Data — (ZIP) [file pone.0218114.s008.zip › Raw data/Characteristics/SEM/+1/2-1-3.bmp]

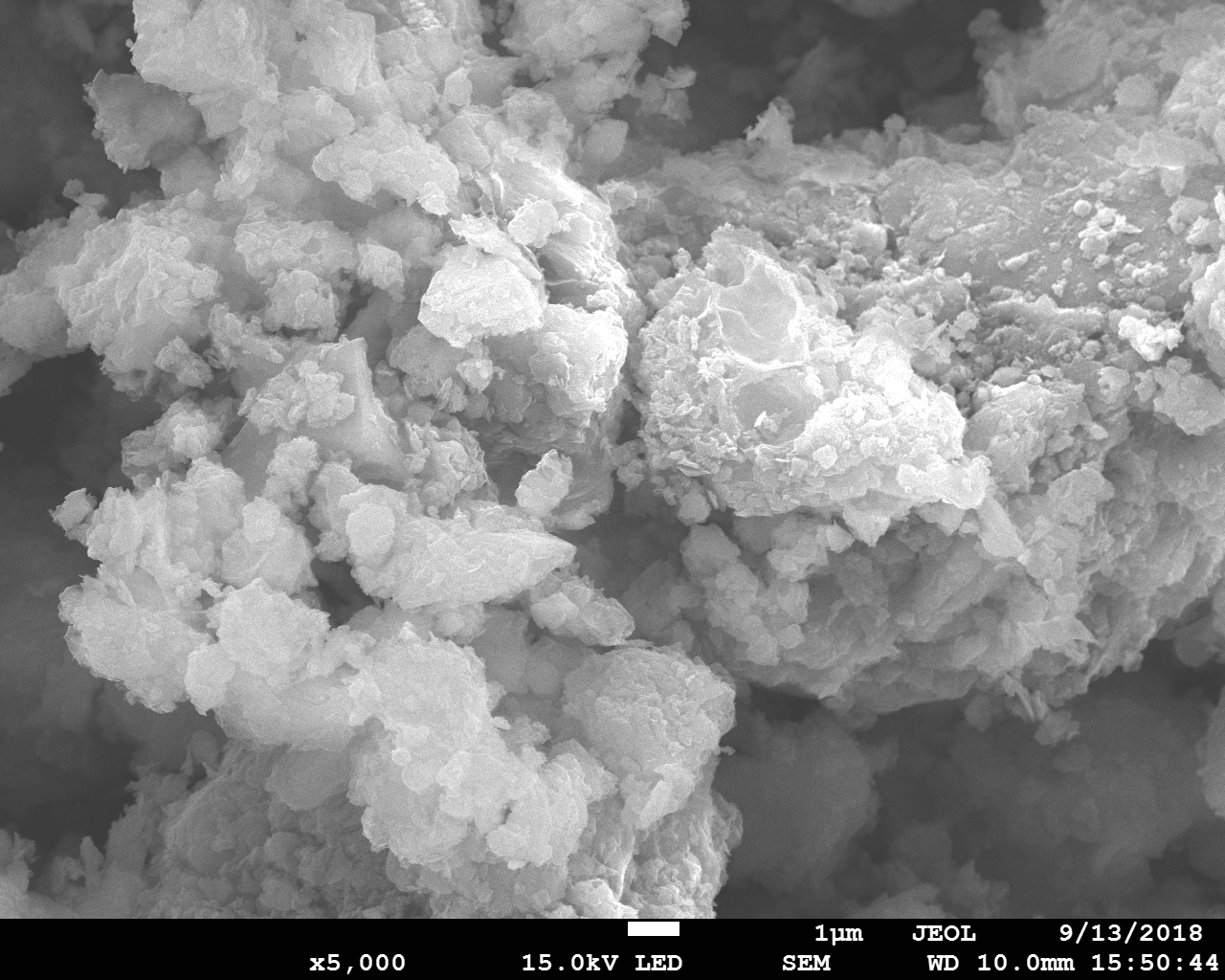

Supplement: S1 Data — (ZIP) [file pone.0218114.s008.zip › Raw data/Characteristics/SEM/+1/2-1-4.bmp]

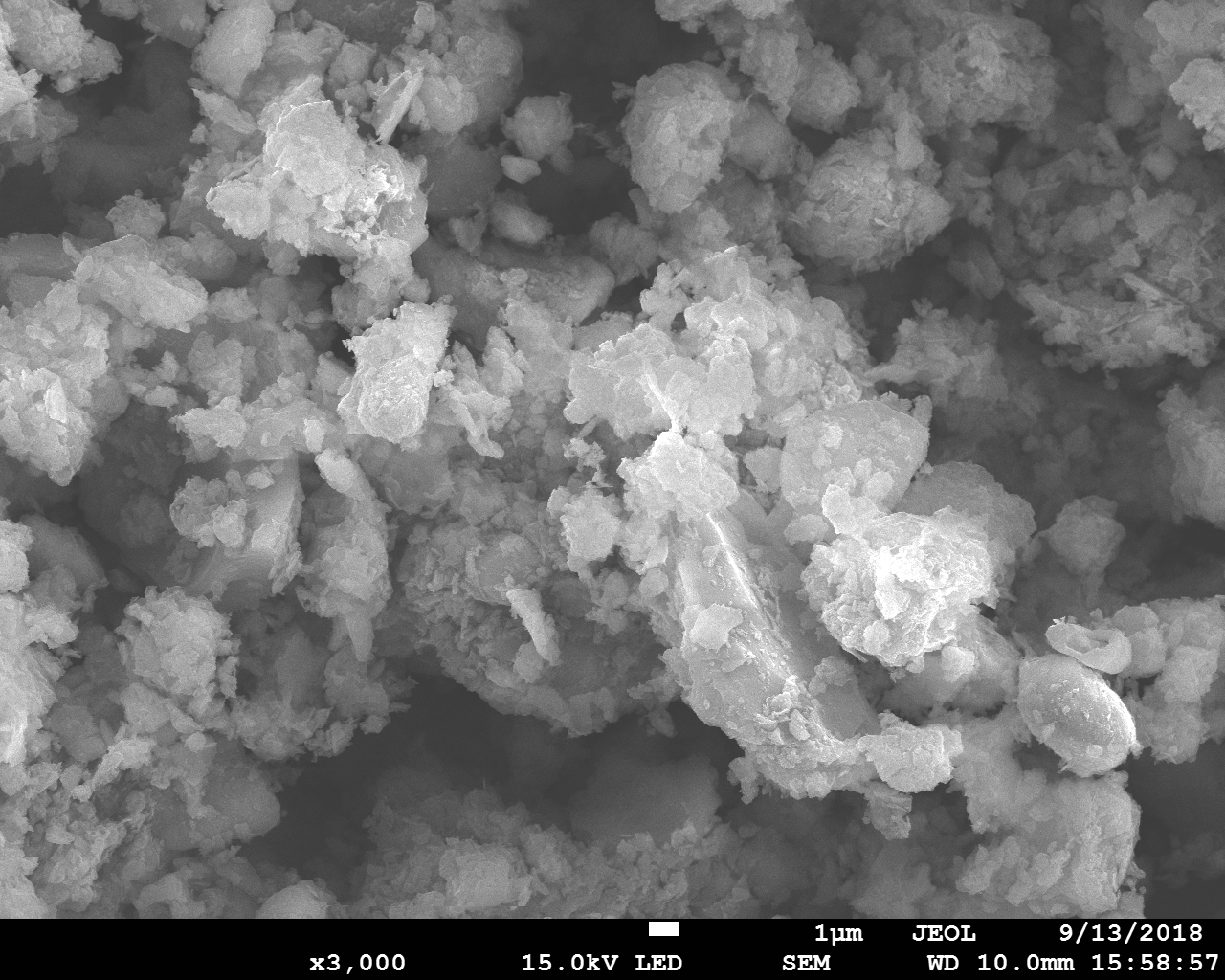

Supplement: S1 Data — (ZIP) [file pone.0218114.s008.zip › Raw data/Characteristics/SEM/+1/2-2-1.bmp]

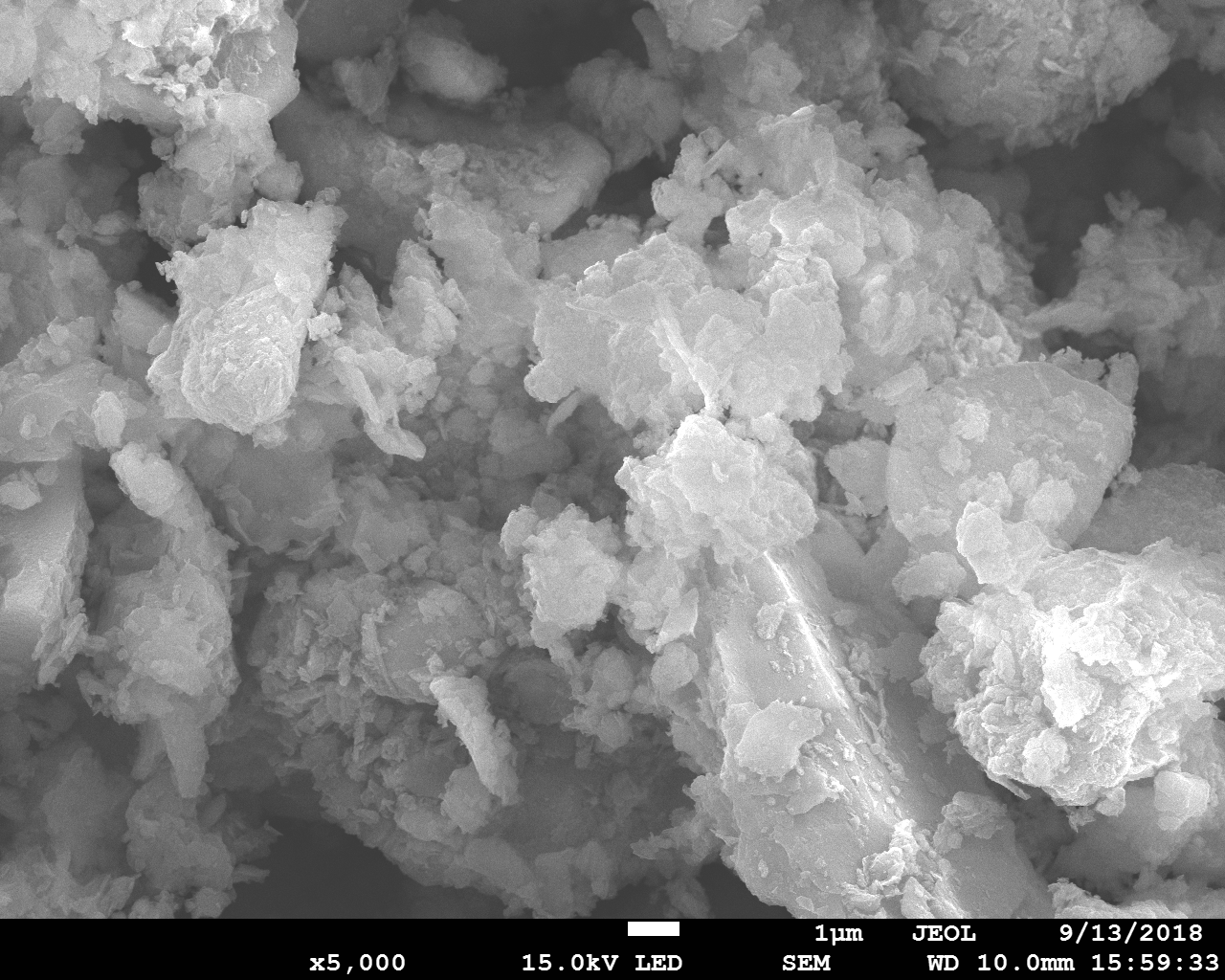

Supplement: S1 Data — (ZIP) [file pone.0218114.s008.zip › Raw data/Characteristics/SEM/+1/2-2-2.bmp]

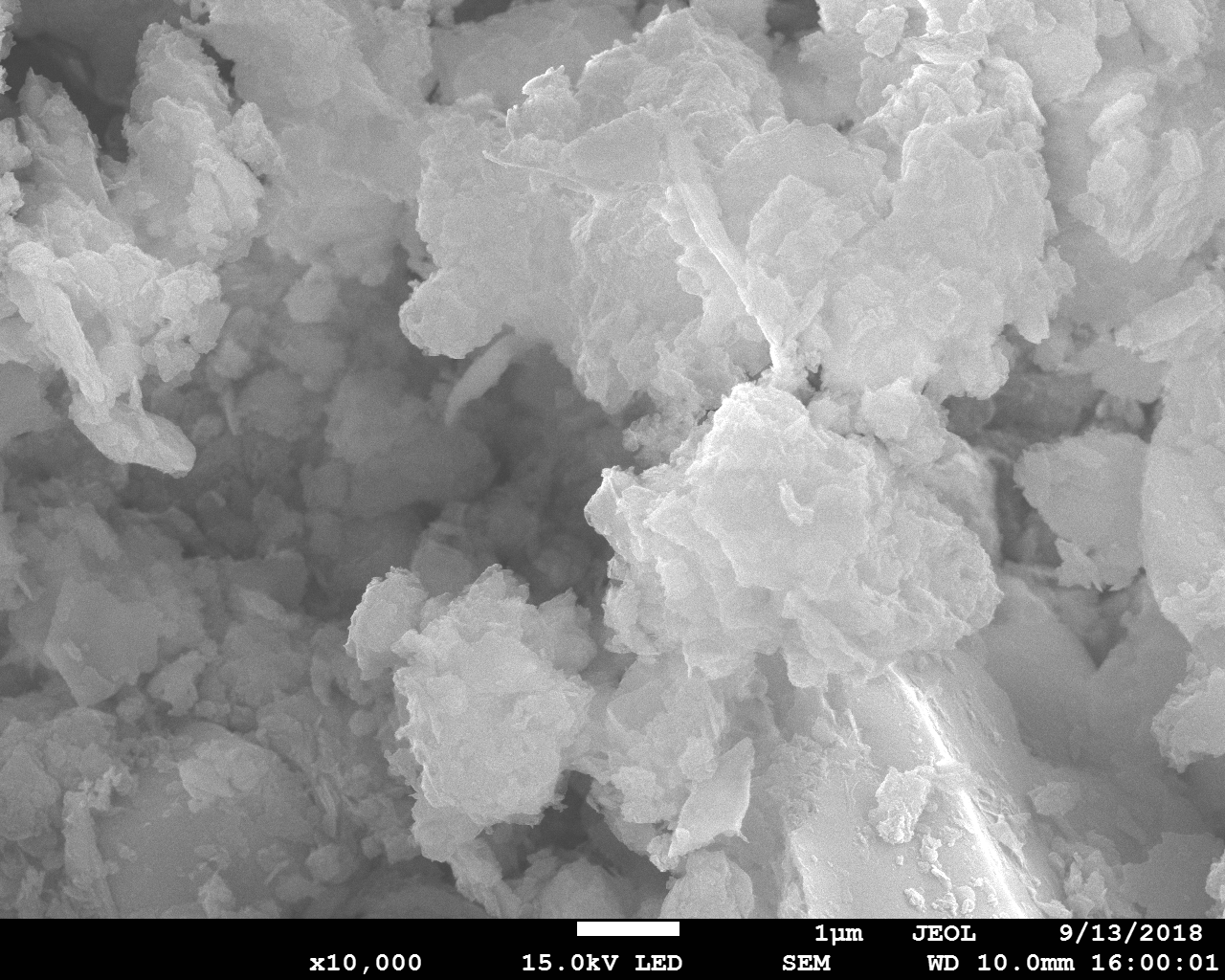

Supplement: S1 Data — (ZIP) [file pone.0218114.s008.zip › Raw data/Characteristics/SEM/+1/2-2-3.bmp]

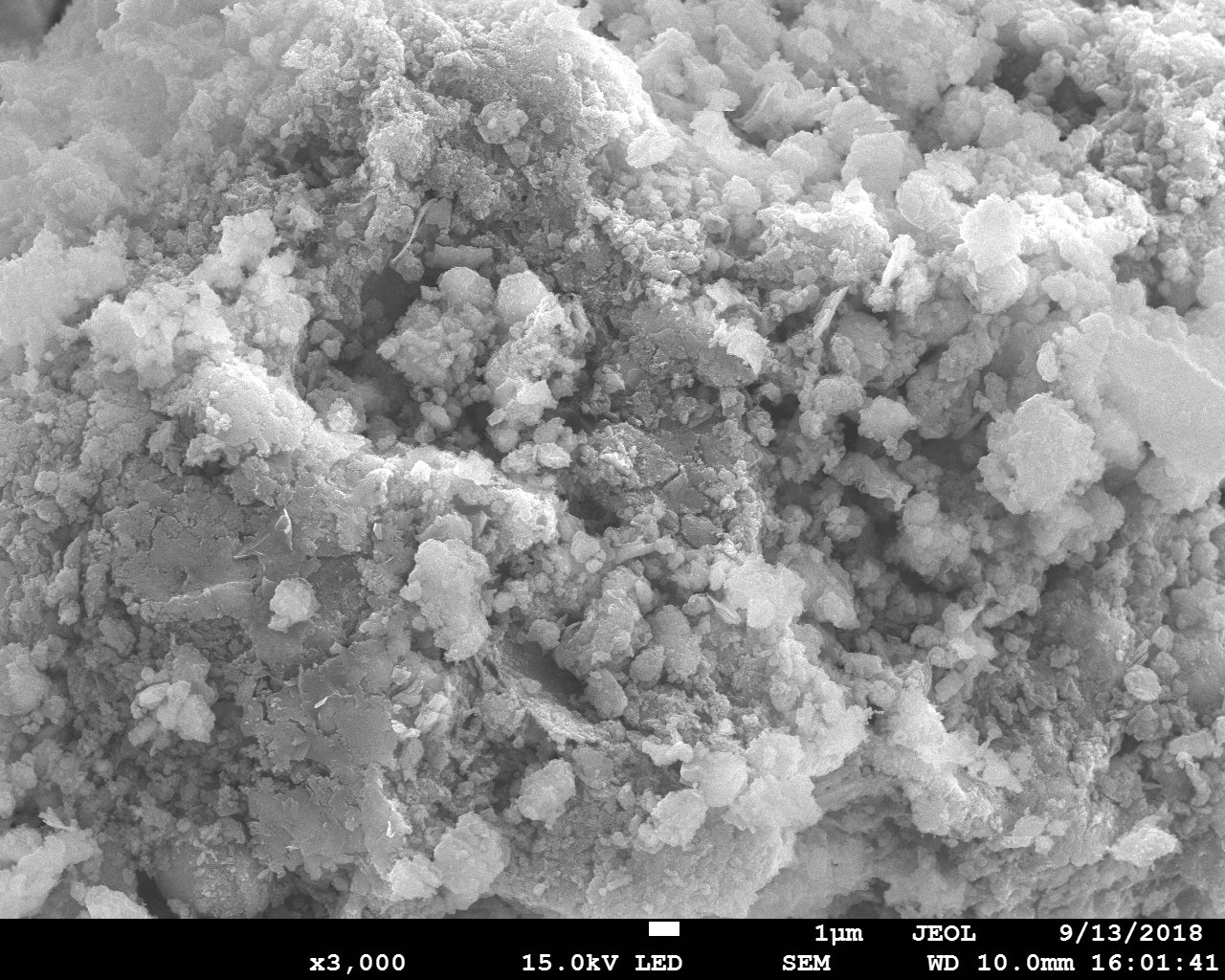

Supplement: S1 Data — (ZIP) [file pone.0218114.s008.zip › Raw data/Characteristics/SEM/+1/3-1-1.bmp]

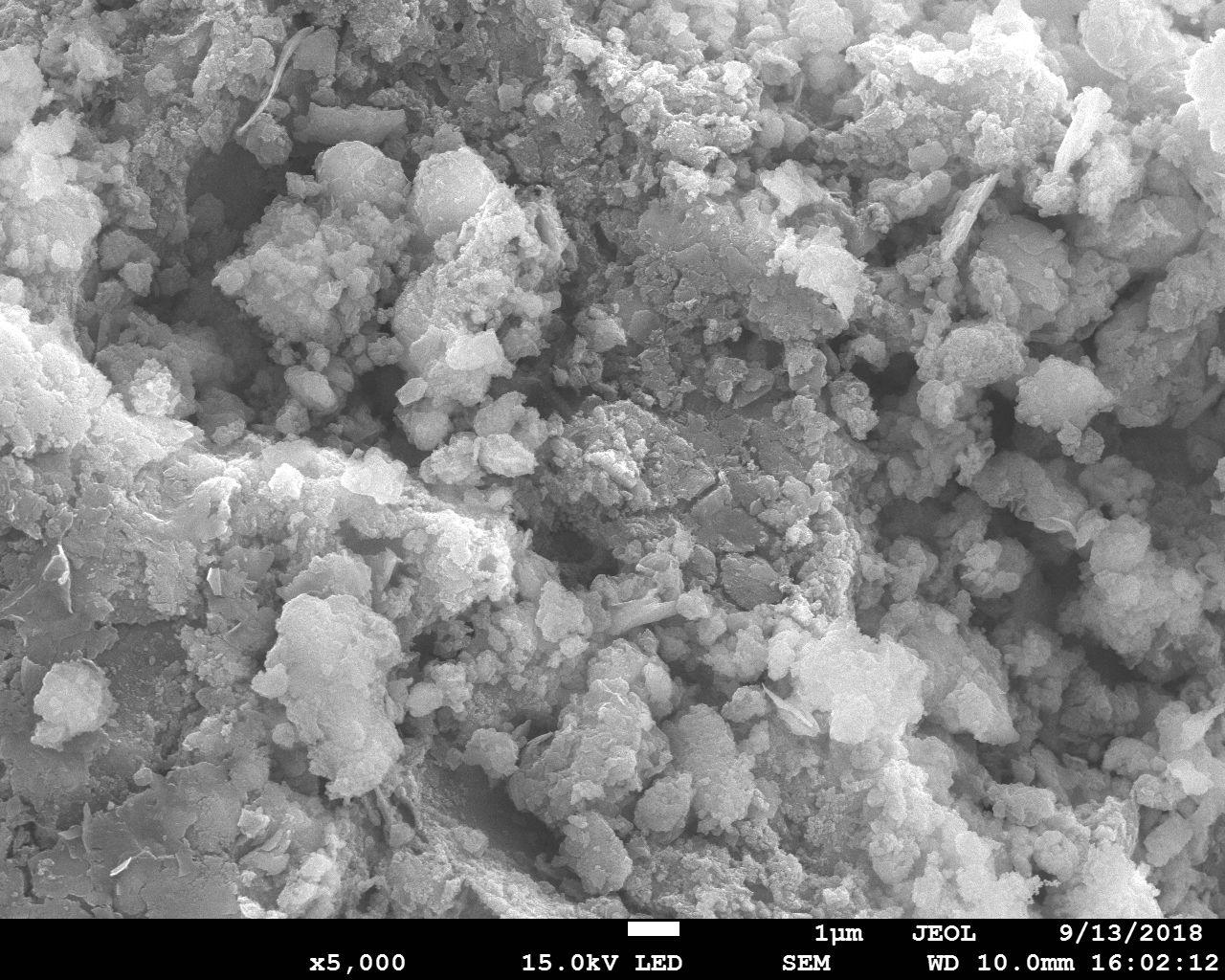

Supplement: S1 Data — (ZIP) [file pone.0218114.s008.zip › Raw data/Characteristics/SEM/+1/3-1-2.bmp]

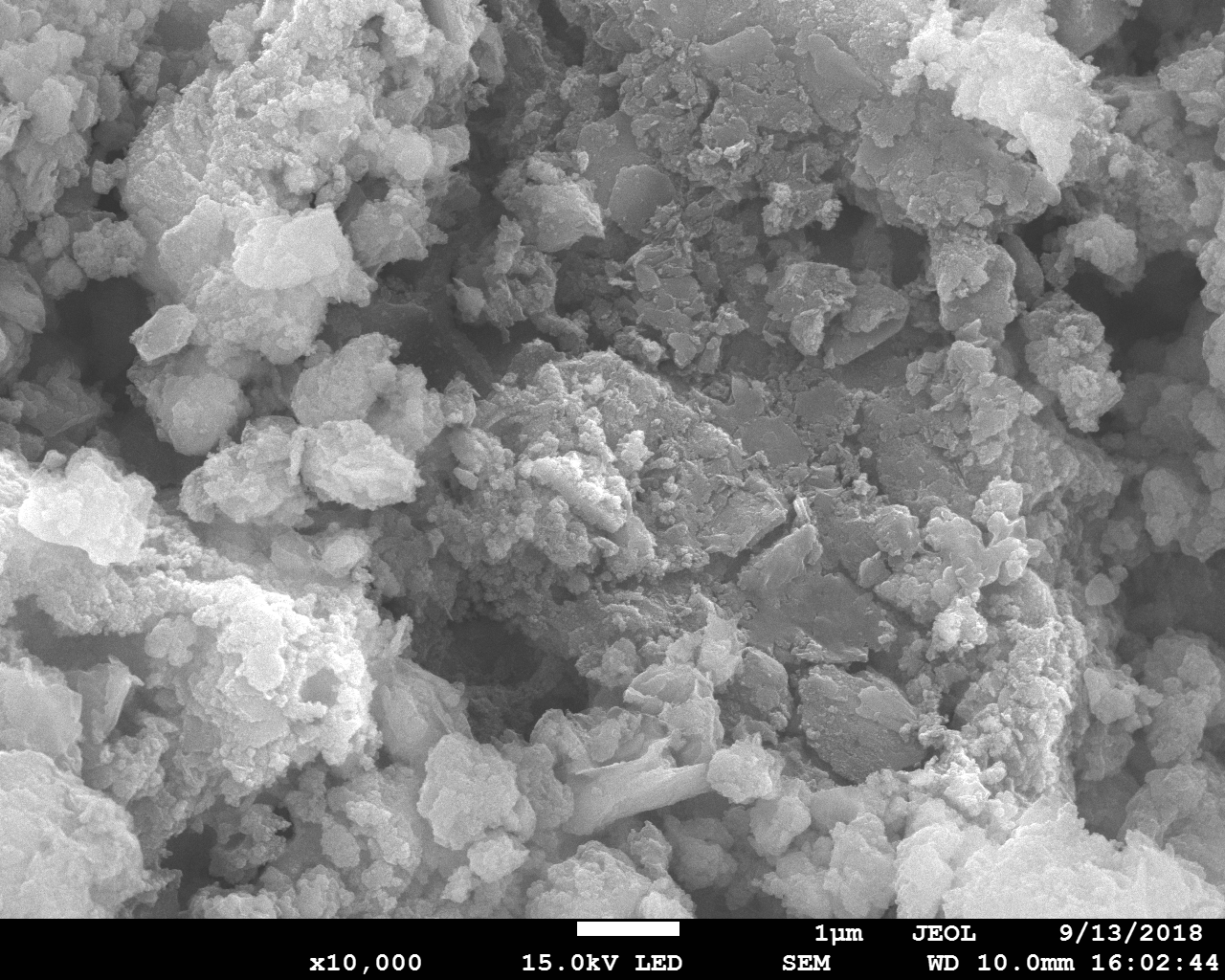

Supplement: S1 Data — (ZIP) [file pone.0218114.s008.zip › Raw data/Characteristics/SEM/+1/3-1-3.bmp]

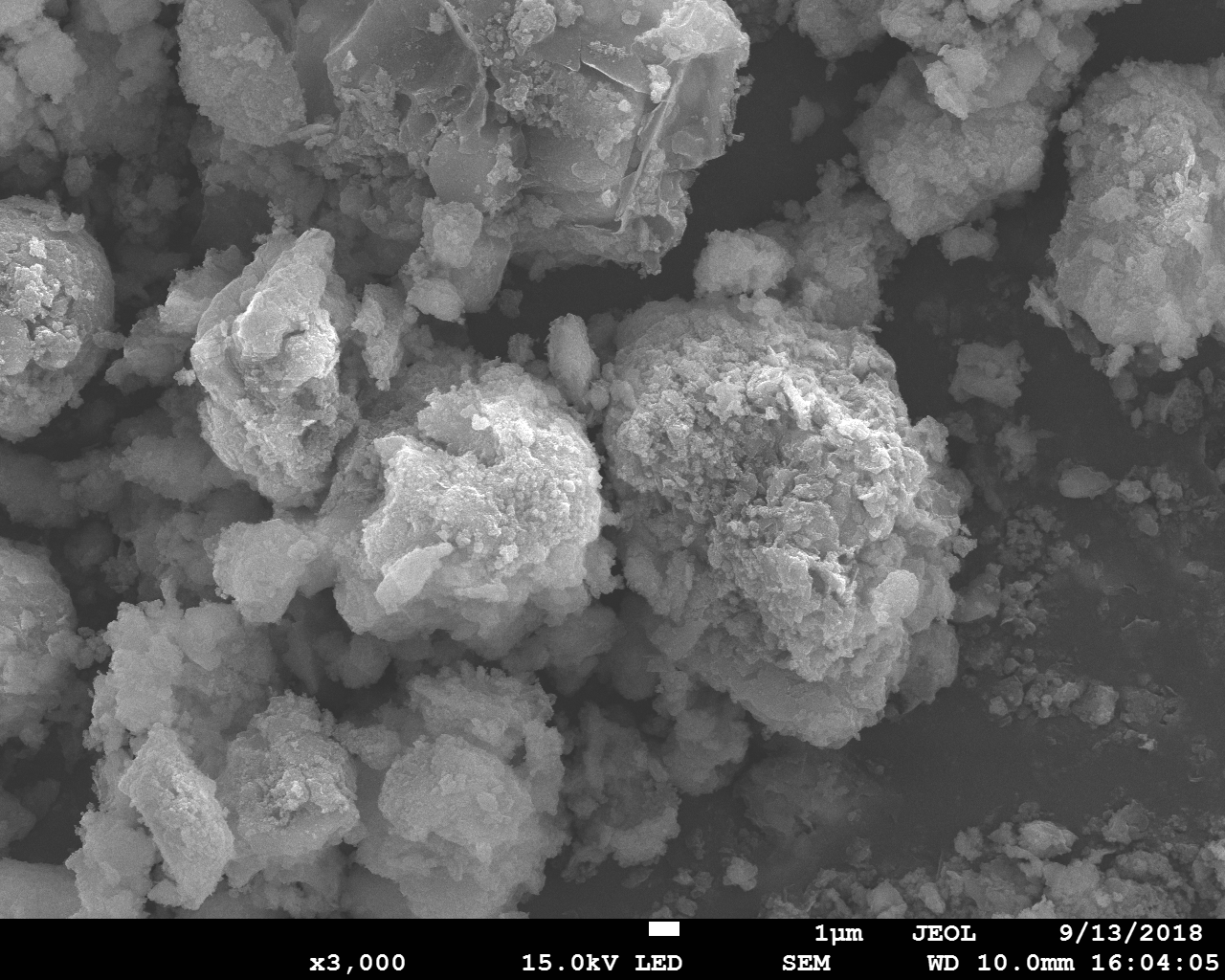

Supplement: S1 Data — (ZIP) [file pone.0218114.s008.zip › Raw data/Characteristics/SEM/+1/3-2-1.bmp]

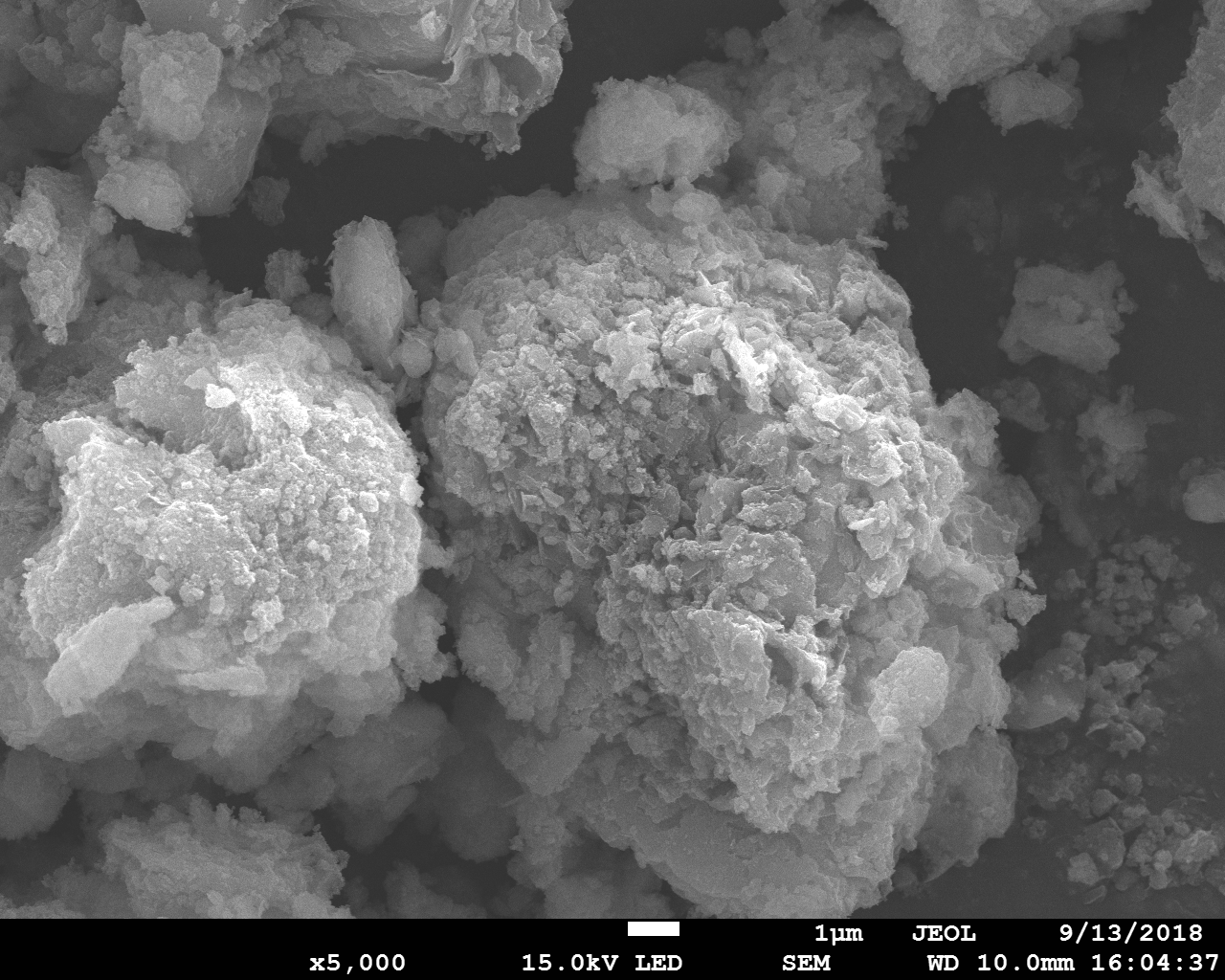

Supplement: S1 Data — (ZIP) [file pone.0218114.s008.zip › Raw data/Characteristics/SEM/+1/3-2-2.bmp]

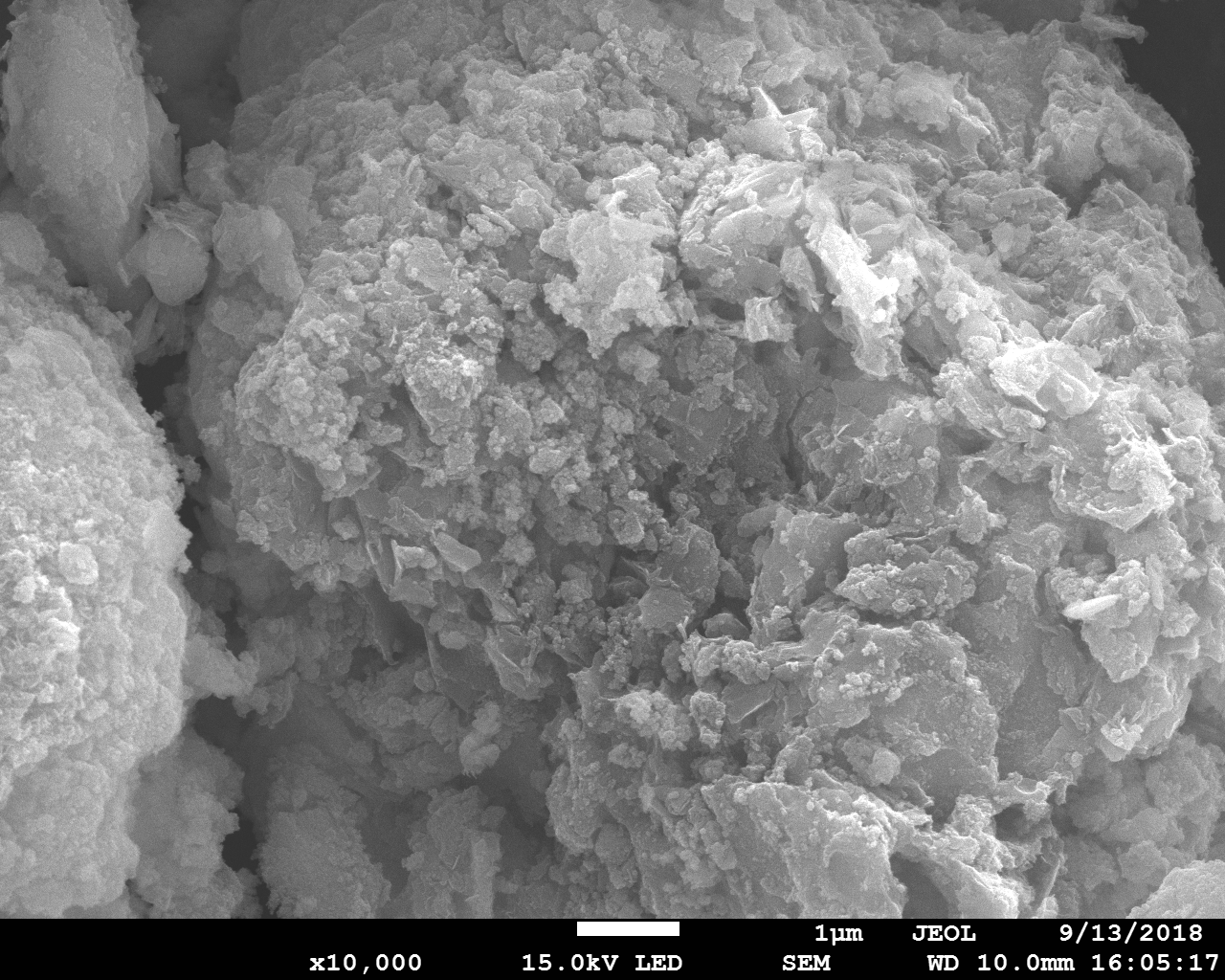

Supplement: S1 Data — (ZIP) [file pone.0218114.s008.zip › Raw data/Characteristics/SEM/+1/3-2-3.bmp]

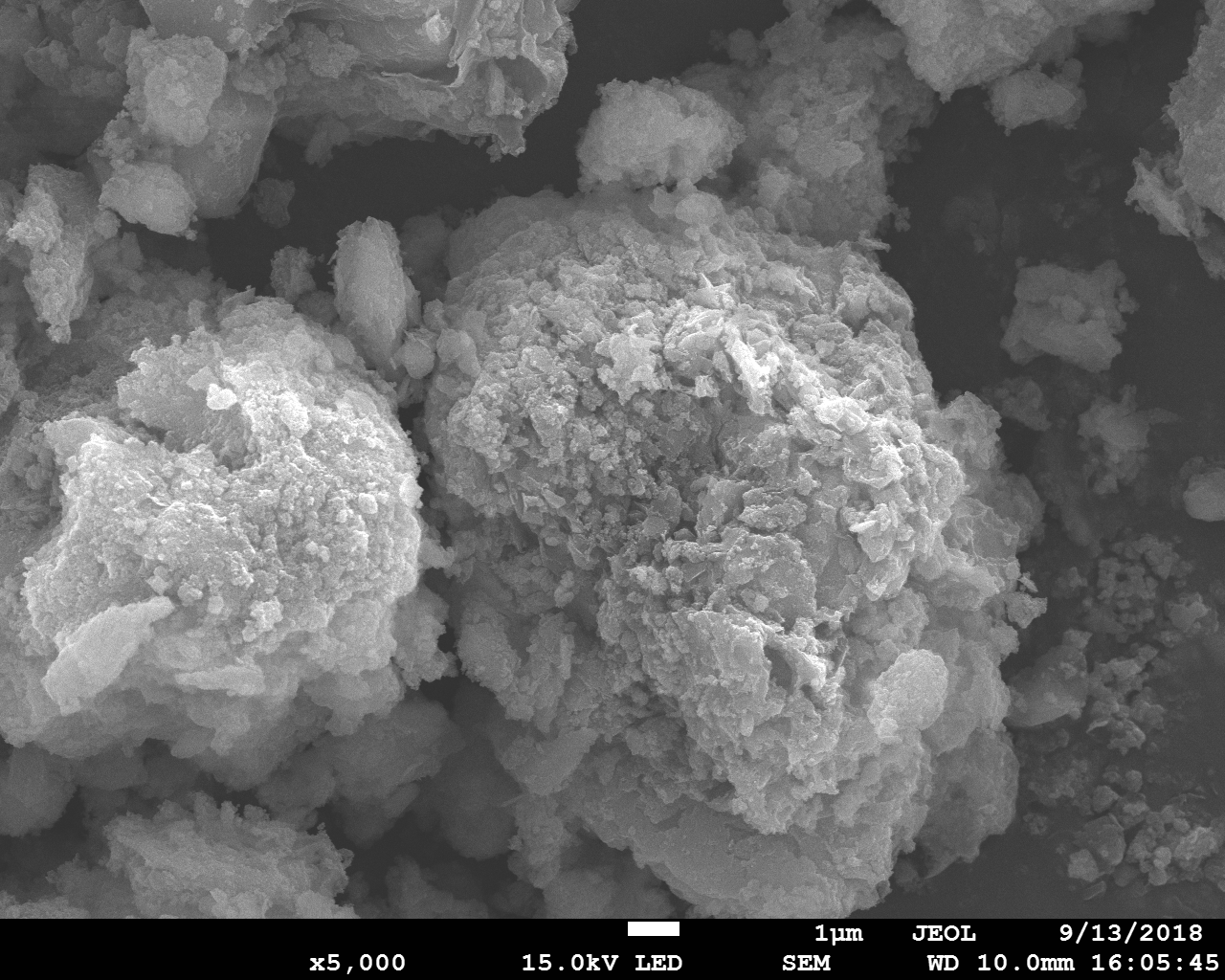

Supplement: S1 Data — (ZIP) [file pone.0218114.s008.zip › Raw data/Characteristics/SEM/+1/3-2-4.bmp]

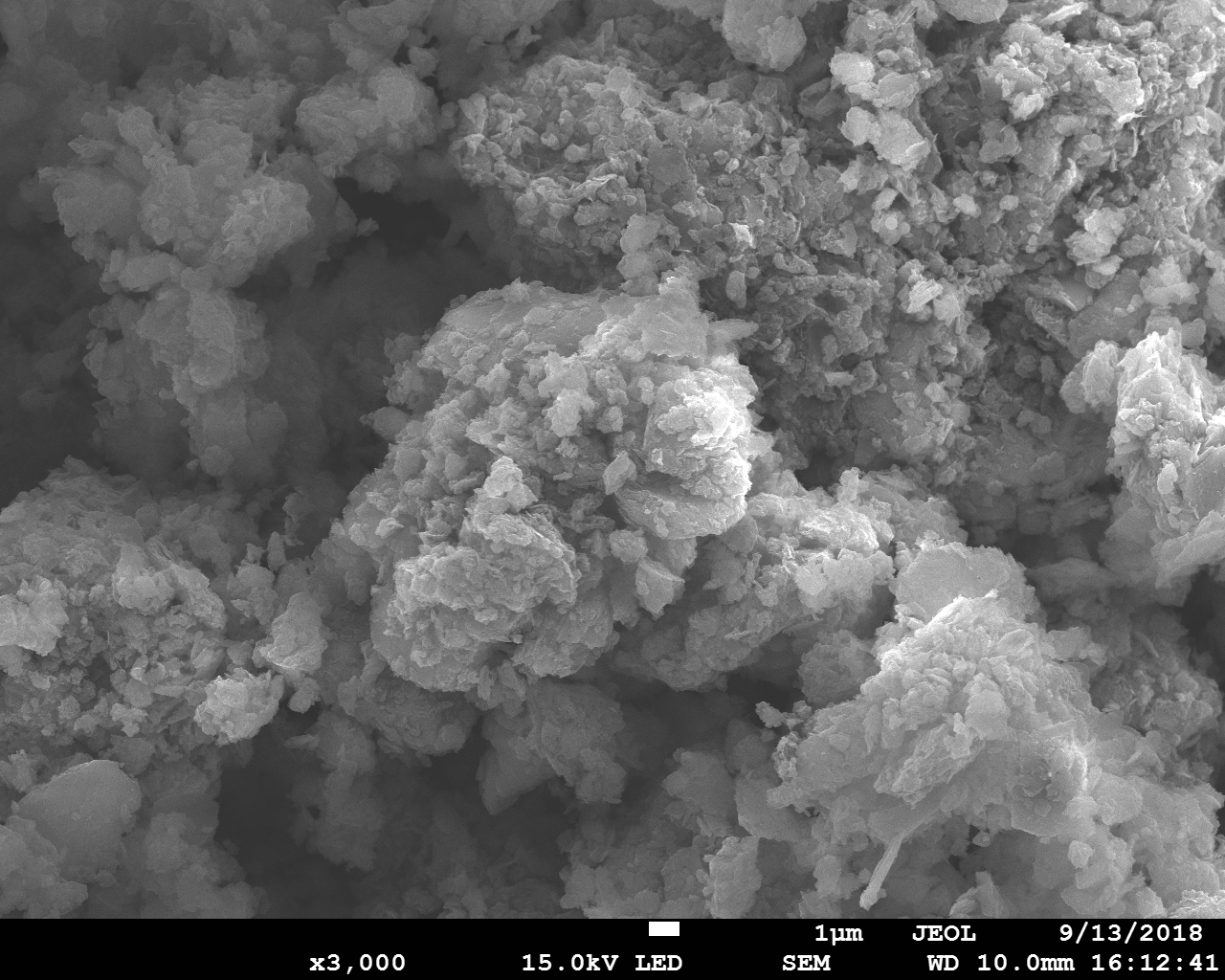

Supplement: S1 Data — (ZIP) [file pone.0218114.s008.zip › Raw data/Characteristics/SEM/+1/4-1-1.bmp]

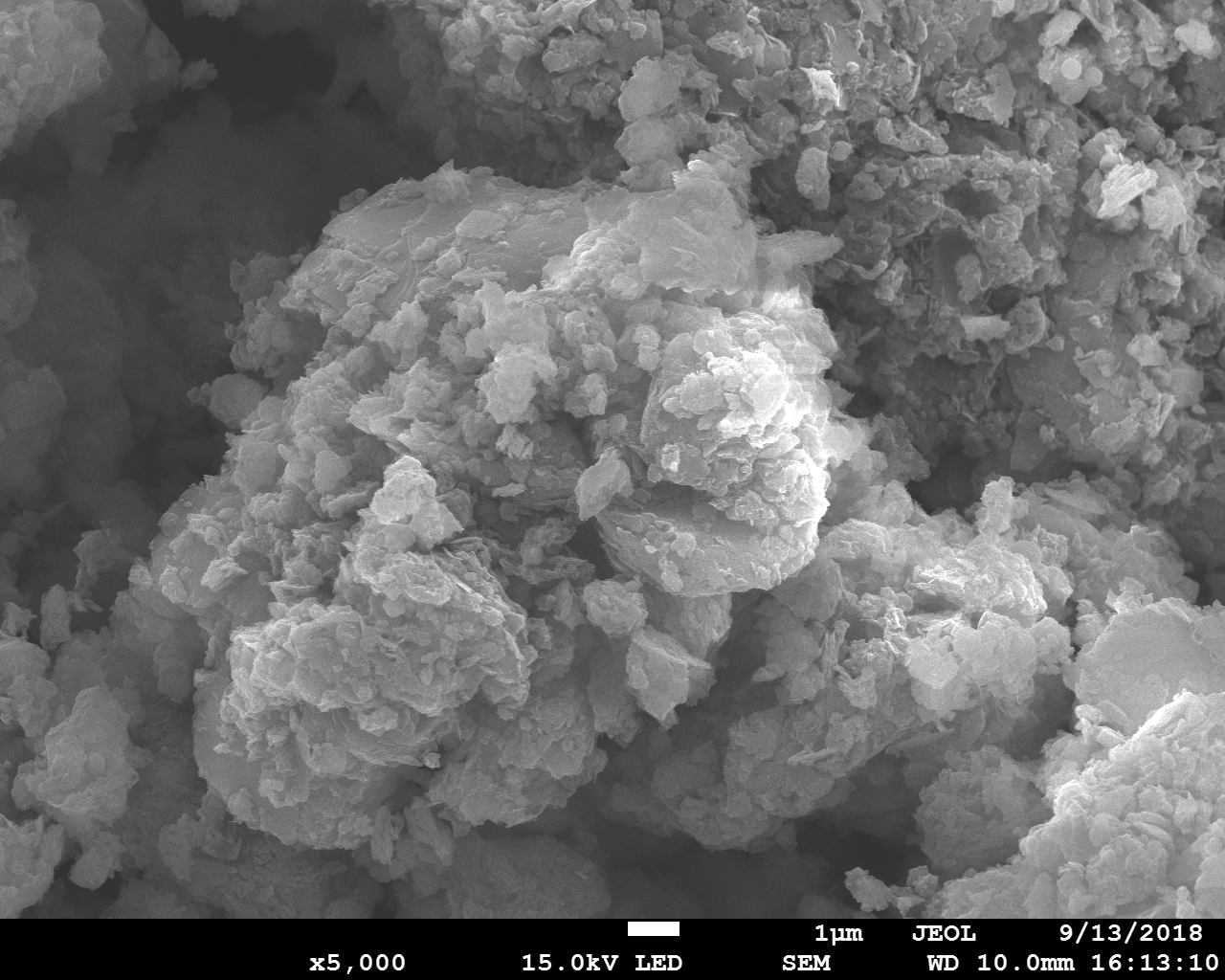

Supplement: S1 Data — (ZIP) [file pone.0218114.s008.zip › Raw data/Characteristics/SEM/+1/4-1-2.bmp]

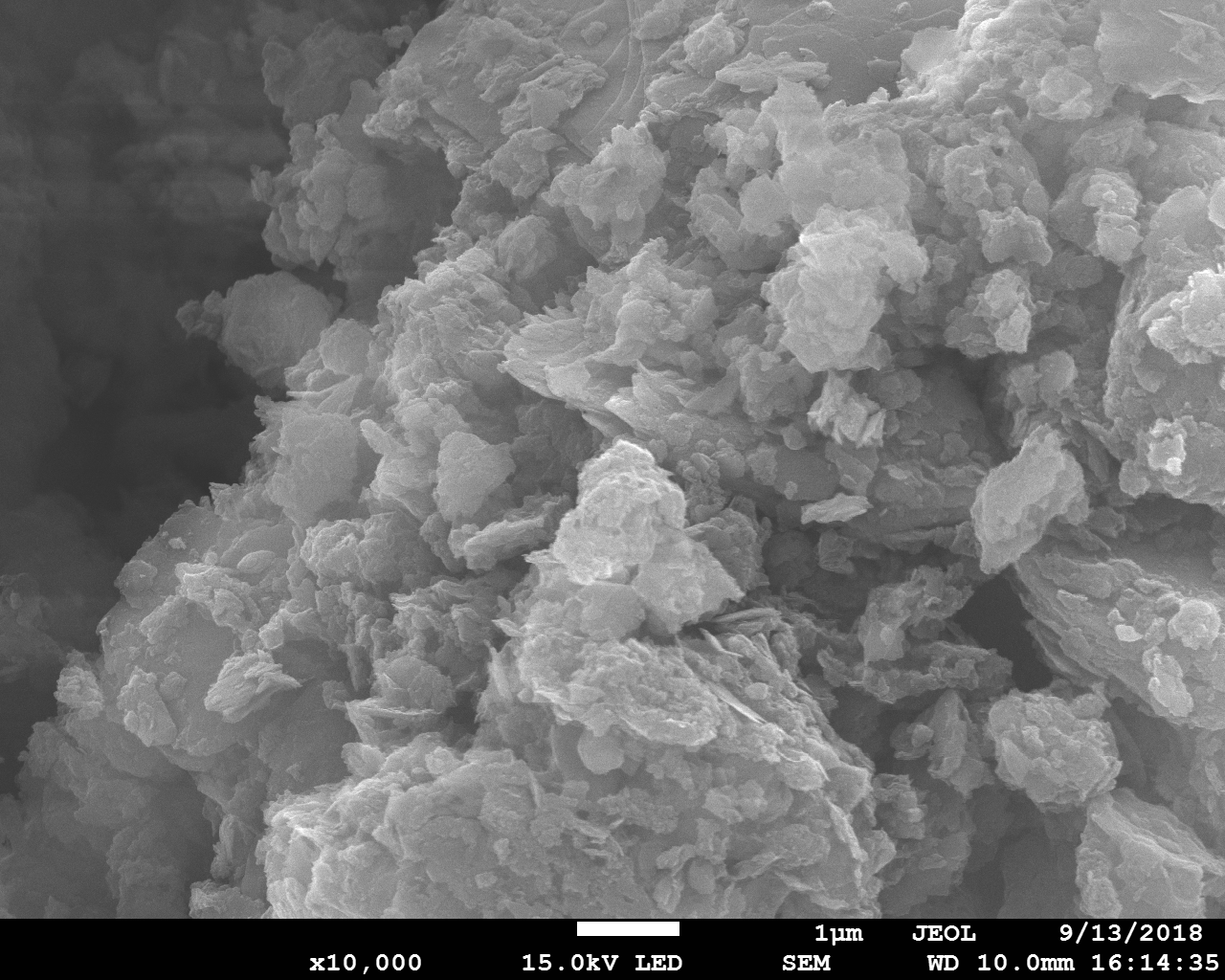

Supplement: S1 Data — (ZIP) [file pone.0218114.s008.zip › Raw data/Characteristics/SEM/+1/4-1-3.bmp]

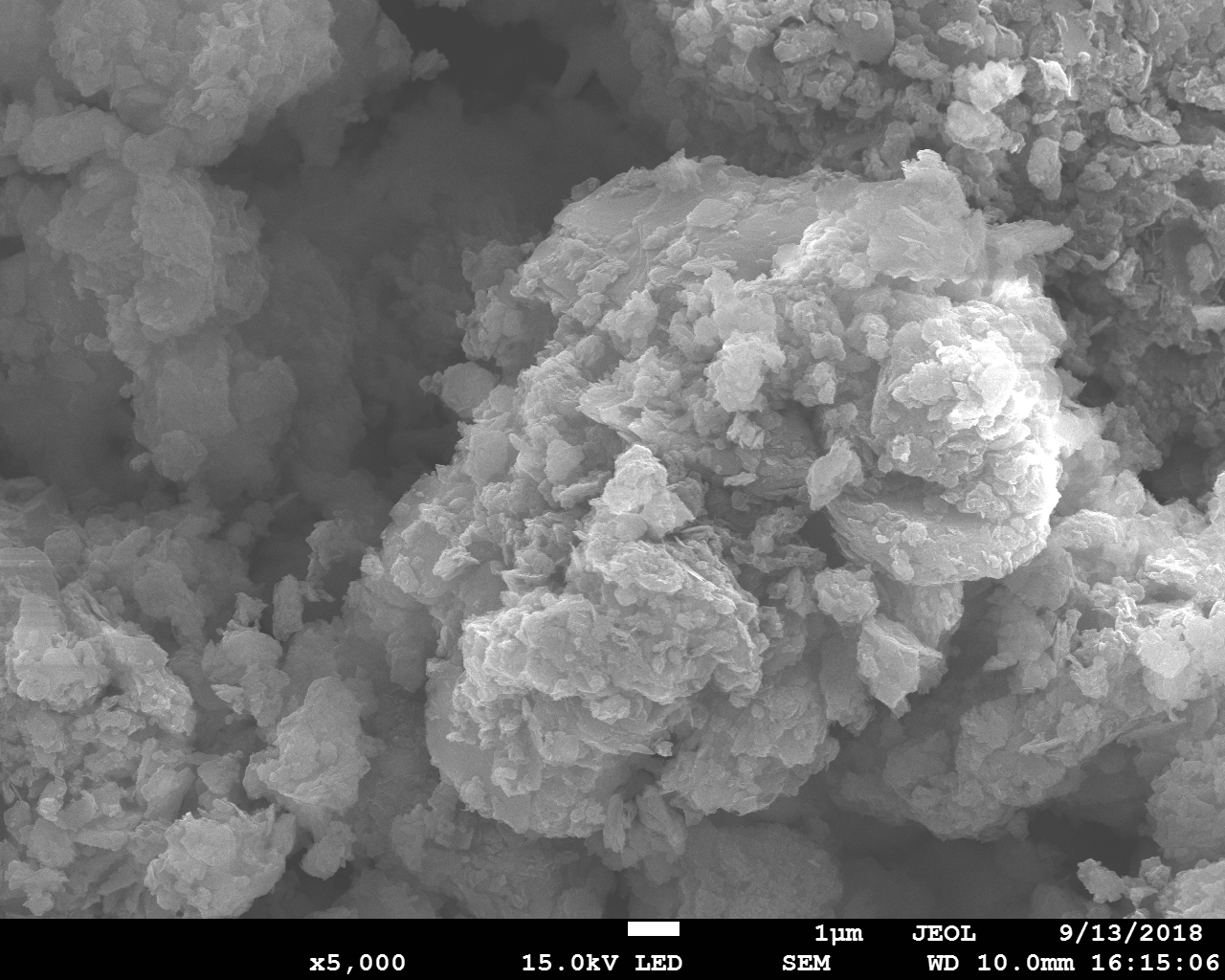

Supplement: S1 Data — (ZIP) [file pone.0218114.s008.zip › Raw data/Characteristics/SEM/+1/4-1-4.bmp]

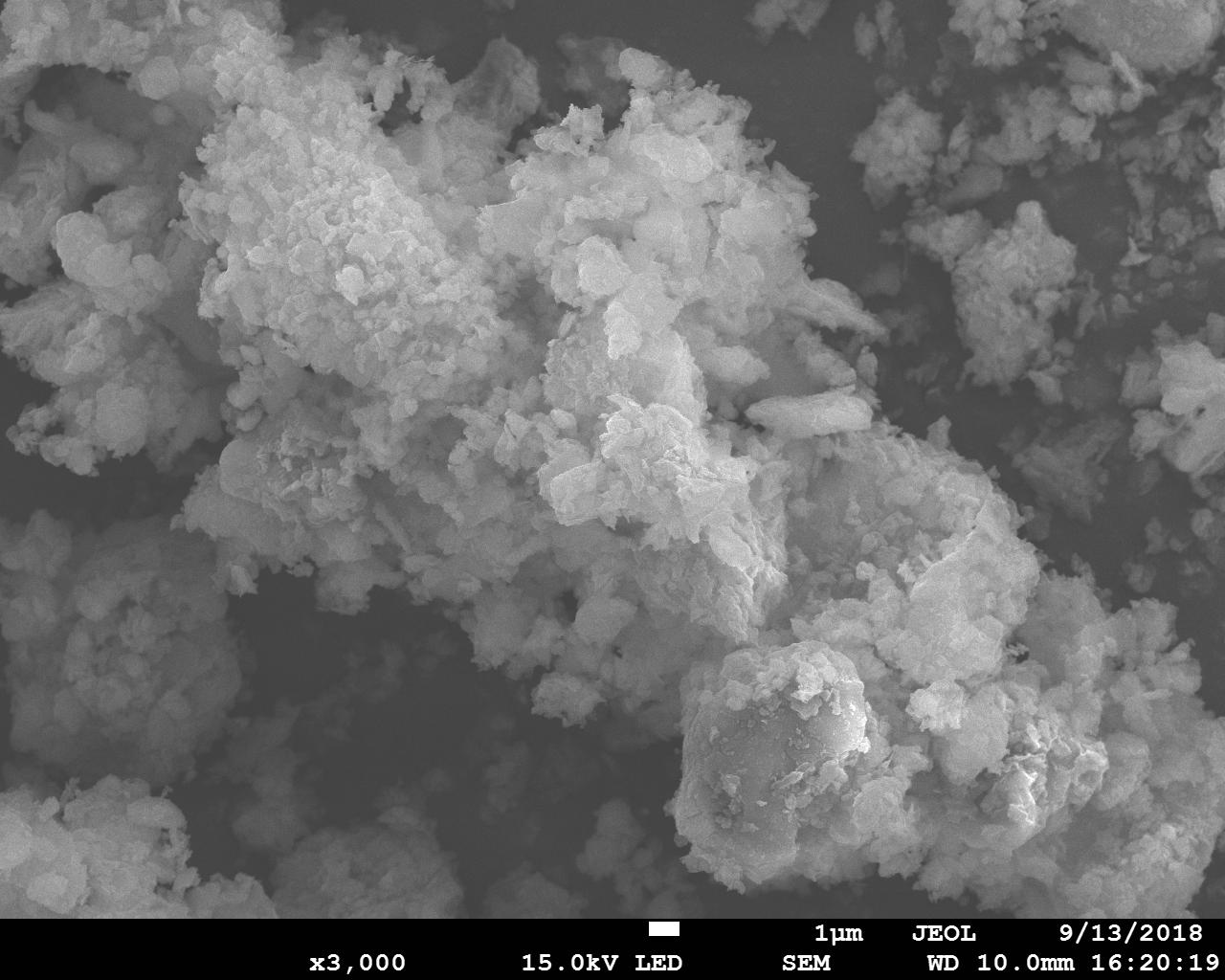

Supplement: S1 Data — (ZIP) [file pone.0218114.s008.zip › Raw data/Characteristics/SEM/+1/4-2-1.bmp]

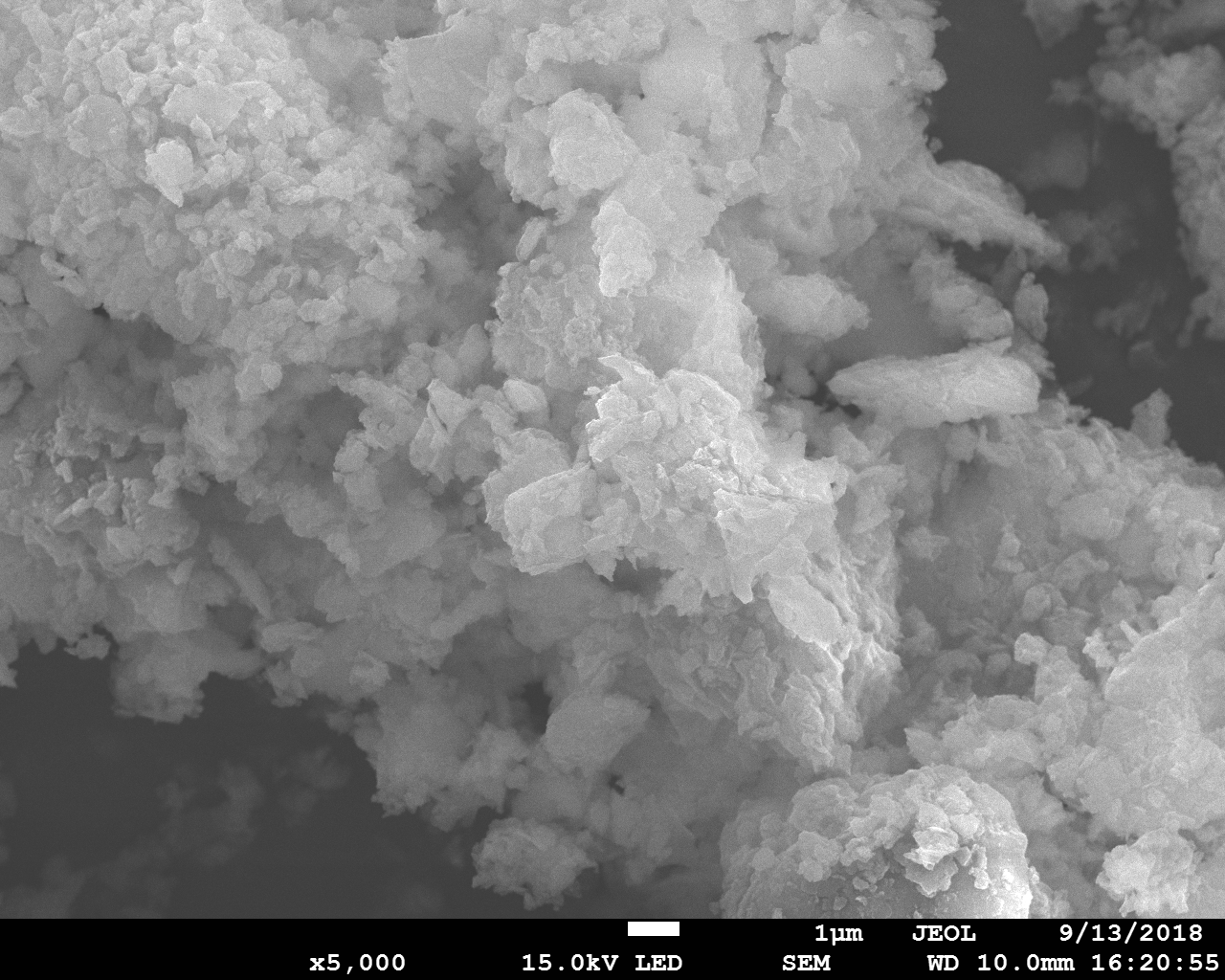

Supplement: S1 Data — (ZIP) [file pone.0218114.s008.zip › Raw data/Characteristics/SEM/+1/4-2-2.bmp]

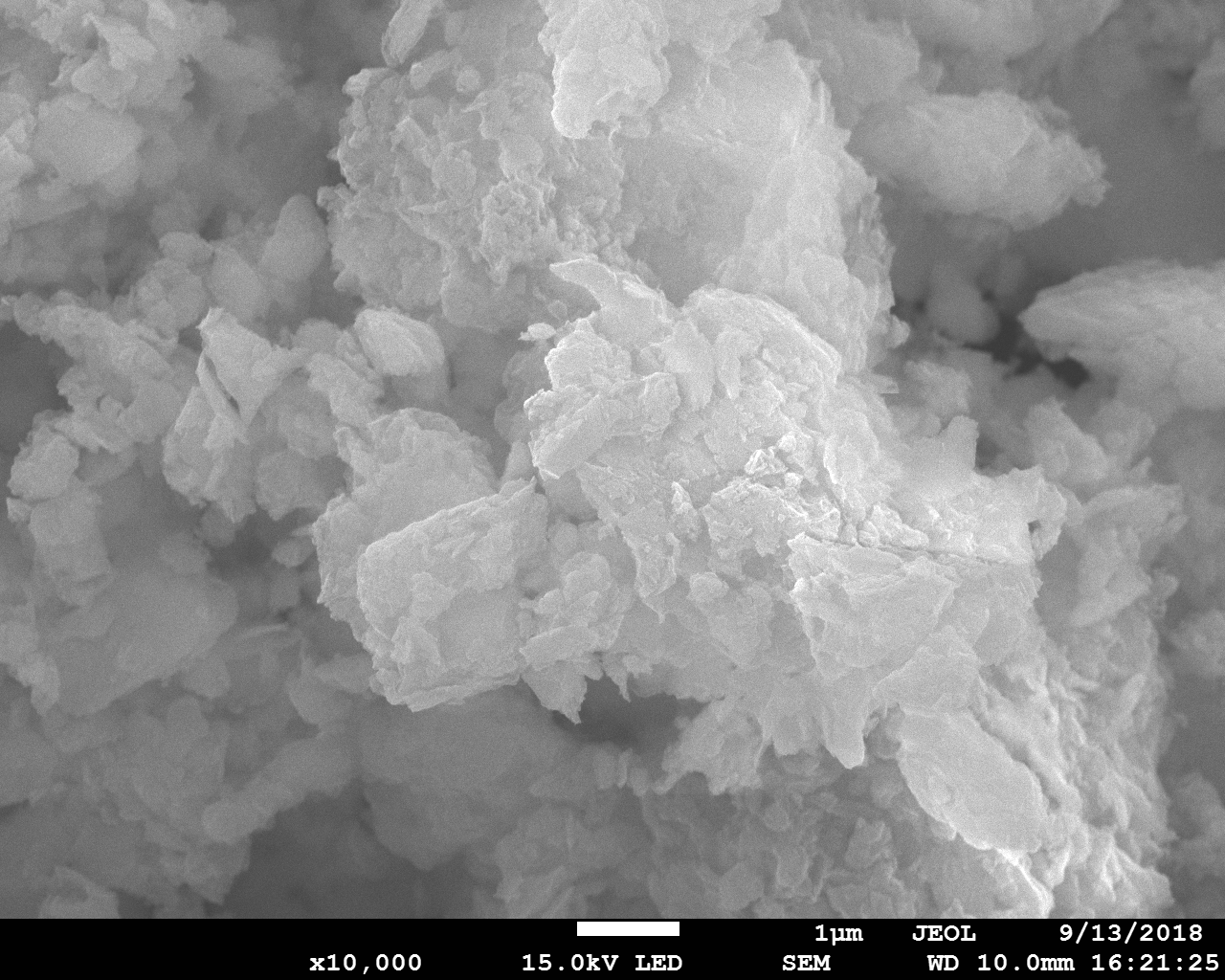

Supplement: S1 Data — (ZIP) [file pone.0218114.s008.zip › Raw data/Characteristics/SEM/+1/4-2-3.bmp]

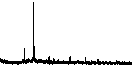

Supplement: S1 Data — (ZIP) [file pone.0218114.s008.zip › Raw data/Characteristics/XRD/20180913dxh/M-1.jip]

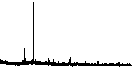

Supplement: S1 Data — (ZIP) [file pone.0218114.s008.zip › Raw data/Characteristics/XRD/20180913dxh/M-2.jip]

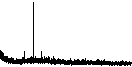

Supplement: S1 Data — (ZIP) [file pone.0218114.s008.zip › Raw data/Characteristics/XRD/20180913dxh/S-1.jip]

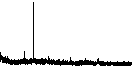

Supplement: S1 Data — (ZIP) [file pone.0218114.s008.zip › Raw data/Characteristics/XRD/20180913dxh/S-2.jip]
