# Supplementary material for: Highly efficient nickel (II) removal by sewage sludge biochar supported α-Fe2O3 and α-FeOOH: Sorption characteristics and mechanisms
Source: PLoS One. 2019 Jun 12;14(6):e0218114. doi: 10.1371/journal.pone.0218114 (PMC6561682; doi:10.1371/journal.pone.0218114)
Supplement: S1 Data — (ZIP) [file pone.0218114.s008.zip › Raw data/Characteristics/EDS/Data/reports/1_2018-09-13_16-30-14.docx]

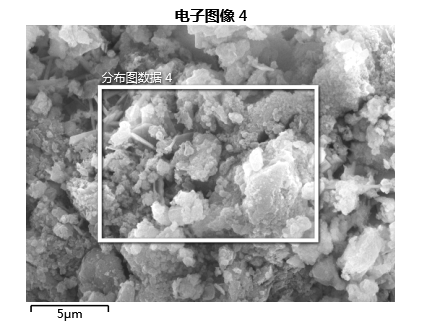

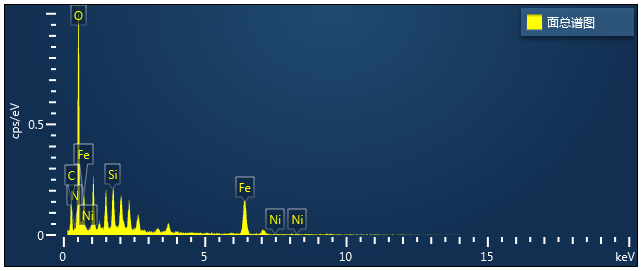


|  |  |  |  |  |  |  |  |
| --- | --- | --- | --- | --- | --- | --- | --- |
| Element | Line type | Concentration | Revision | k ratio | wt% | wt% Sigma | Molecular % |
| C | K | 1.47 | 0.28 | 0.01466 | 17.56 | 0.96 | 29.34 |
| N | K | 0.10 | 1.47 | 0.00018 | 0.24 | 1.01 | 0.34 |
| O | K | 17.48 | 1.36 | 0.05884 | 43.36 | 0.96 | 54.38 |
| Si | K | 1.45 | 0.87 | 0.01153 | 5.63 | 0.26 | 4.02 |
| Fe | K | 8.18 | 0.84 | 0.08179 | 32.98 | 0.91 | 11.85 |
| Ni | K | 0.05 | 0.81 | 0.00054 | 0.22 | 0.45 | 0.08 |
| Total |  |  |  |  | 100.00 |  |  |

100.00
