# Supplementary material for: Highly efficient nickel (II) removal by sewage sludge biochar supported α-Fe2O3 and α-FeOOH: Sorption characteristics and mechanisms
Source: PLoS One. 2019 Jun 12;14(6):e0218114. doi: 10.1371/journal.pone.0218114 (PMC6561682; doi:10.1371/journal.pone.0218114)
Supplement: S1 Data — (ZIP) [file pone.0218114.s008.zip › Raw data/Characteristics/EDS/Data/reports/2_2018-09-13_15-59-51.docx]

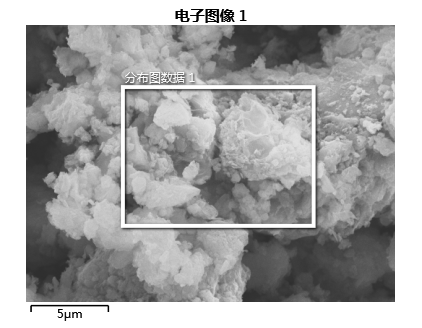

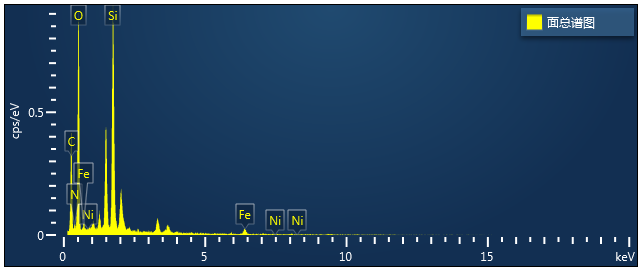


| Element | Line type | Concentration | Revision | k ratio | wt% | wt% Sigma | Molecular % |
| --- | --- | --- | --- | --- | --- | --- | --- |
| C | K | 3.66 | 0.25 | 0.03662 | 34.00 | 0.92 | 44.06 |
| N | K | 1.38 | 0.86 | 0.00245 | 3.78 | 1.18 | 4.20 |
| O | K | 14.72 | 0.80 | 0.04954 | 43.28 | 0.89 | 42.10 |
| Si | K | 6.60 | 0.98 | 0.05230 | 15.81 | 0.37 | 8.76 |
| Fe | K | 1.03 | 0.78 | 0.01030 | 3.13 | 0.31 | 0.87 |
| Ni | K | 0.00 | 0.77 | 0.00000 | 0.00 | 0.00 | 0.00 |
| Total: |  |  |  |  | 100.00 |  | 100.00 |
