# Supplementary material for: Highly efficient nickel (II) removal by sewage sludge biochar supported α-Fe2O3 and α-FeOOH: Sorption characteristics and mechanisms
Source: PLoS One. 2019 Jun 12;14(6):e0218114. doi: 10.1371/journal.pone.0218114 (PMC6561682; doi:10.1371/journal.pone.0218114)
Supplement: S1 Data — (ZIP) [file pone.0218114.s008.zip › Raw data/Characteristics/EDS/Data/reports/3_2018-09-13_16-13-01.docx]

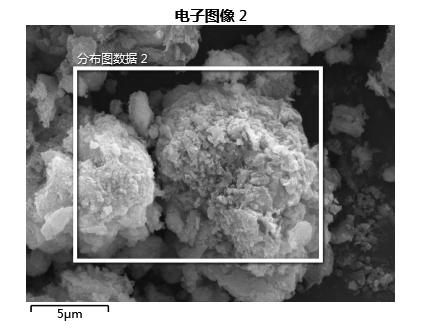

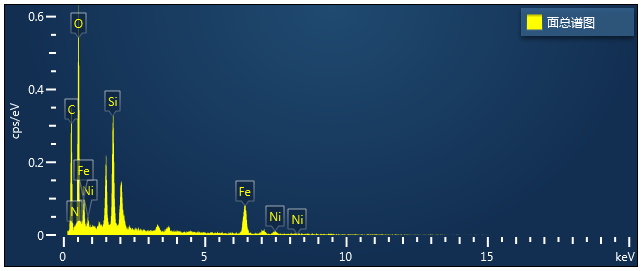


| Element | Line type | Concentration | Revision | k ratio | wt% | wt% Sigma | Molecular % |
| --- | --- | --- | --- | --- | --- | --- | --- |
| C | K | 3.69 | 0.30 | 0.03692 | 34.44 | 0.95 | 48.51 |
| N | K | 0.66 | 0.93 | 0.00118 | 2.00 | 1.20 | 2.42 |
| O | K | 11.65 | 0.90 | 0.03921 | 36.29 | 0.87 | 38.37 |
| Si | K | 2.72 | 0.92 | 0.02153 | 8.25 | 0.27 | 4.97 |
| Fe | K | 4.77 | 0.81 | 0.04768 | 16.50 | 0.65 | 5.00 |
| Ni | K | 0.71 | 0.79 | 0.00710 | 2.51 | 0.48 | 0.72 |
| Total: |  |  |  |  | 100.00 |  | 100.00 |
