# Supplementary material for: Highly efficient nickel (II) removal by sewage sludge biochar supported α-Fe2O3 and α-FeOOH: Sorption characteristics and mechanisms
Source: PLoS One. 2019 Jun 12;14(6):e0218114. doi: 10.1371/journal.pone.0218114 (PMC6561682; doi:10.1371/journal.pone.0218114)
Supplement: S1 Data — (ZIP) [file pone.0218114.s008.zip › Raw data/Characteristics/EDS/Data/reports/4_2018-09-13_16-21-40.docx]

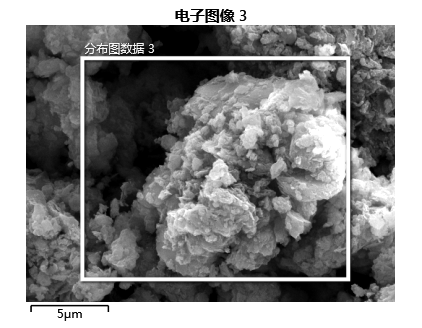

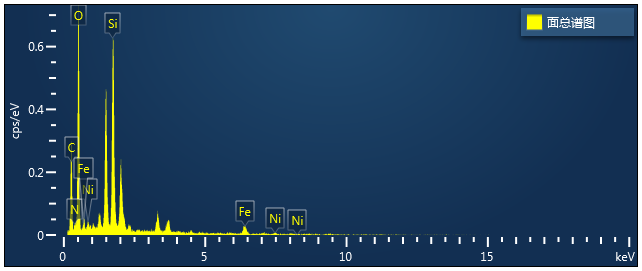


| Element | Line type | Concentration | Revision | k ratio | wt% | wt% Sigma | Molecular % |
| --- | --- | --- | --- | --- | --- | --- | --- |
| C | K | 2.77 | 0.25 | 0.02770 | 32.04 | 1.12 | 42.66 |
| N | K | 1.08 | 0.91 | 0.00193 | 3.50 | 1.43 | 4.00 |
| O | K | 12.39 | 0.84 | 0.04169 | 43.02 | 1.09 | 43.01 |
| Si | K | 4.90 | 0.97 | 0.03884 | 14.86 | 0.44 | 8.47 |
| Fe | K | 1.37 | 0.79 | 0.01370 | 5.10 | 0.47 | 1.46 |
| Ni | K | 0.39 | 0.78 | 0.00392 | 1.47 | 0.45 | 0.40 |
| Total: |  |  |  |  | 100.00 |  | 100.00 |
