# Supplementary material for: Highly efficient nickel (II) removal by sewage sludge biochar supported α-Fe2O3 and α-FeOOH: Sorption characteristics and mechanisms
Source: PLoS One. 2019 Jun 12;14(6):e0218114. doi: 10.1371/journal.pone.0218114 (PMC6561682; doi:10.1371/journal.pone.0218114)
Supplement: S1 Data — (ZIP) [file pone.0218114.s008.zip › Raw data/Characteristics/Figures/FTIR/FTIR-2.pptx]

## Slide 1
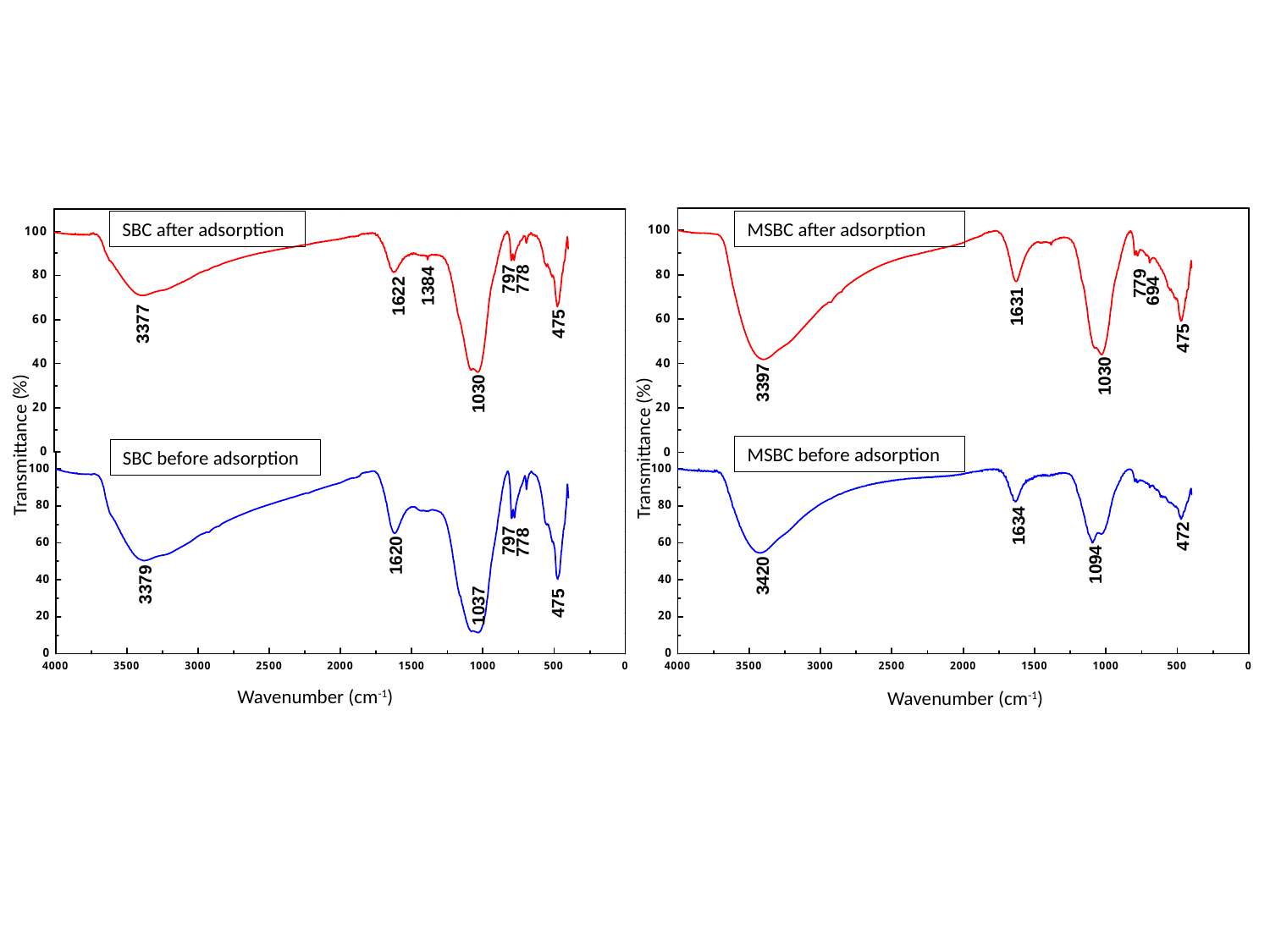

SBC after adsorption
MSBC after adsorption
797
778
779
694
1384
1622
1631
475
3377
475
1030
3397
1030
Transmittance (%)
Transmittance (%)
MSBC before adsorption
SBC before adsorption
1634
472
797
778
1620
1094
3420
3379
475
1037
Wavenumber (cm-1)
Wavenumber (cm-1)
